# Pd- and Cu-catalyzed approaches in the syntheses of new cholane aminoanthraquinone pincer-like ligands

**DOI:** 10.3762/bjoc.13.55

**Published:** 2017-03-20

**Authors:** Nikolay V Lukashev, Gennadii A Grabovyi, Dmitry A Erzunov, Alexey V Kazantsev, Gennadij V Latyshev, Alexei D Averin, Irina P Beletskaya.

**Affiliations:** 1 Department of Chemistry, Lomonosov Moscow State University, Leninskie Gory 1-3, Moscow, 119991, Russia

**Keywords:** amination, aminocholanes, bile acids, cation complexation, Cu-catalysis, diaminoanthraquinone, Pd-catalysis

## Abstract

Cu- and Pd-catalyzed arylation of aminocholanes has been described for the first time. While this Cu-catalyzed protocol provides high yields in reactions of aminocholanes with iodoarenes, Pd catalysis was found to be preferable for the reactions of aminocholanes with dichloroanthraquinones. UV–vis titration of bis(cholanylamino)anthraquinones with a series of cations demonstrated their high binding affinity to Cu^2+^, Al^3+^, and Cr^3+^.

## Introduction

Bile acids are known to ensure vital processes in vertebrate organisms, including metabolism and fat digesting [1–2]. The biological activity of bile acids is mainly based on the properties of their backbone. All bile acids possess a rigid cholane skeleton with two distinct surfaces. The inward hydrophilic surface (with several hydroxy groups) along with the outward hydrophobic face permit bile acids to act as facial amphiphiles [3]. Their carboxylic and hydroxy groups can be easily modified, and the chemistry of these processes has been thoroughly studied [4]. All these options make bile acids the molecules of choice for many applications. The choice of bile acids as template for design of complex molecules has become increasingly popular in pharmacology, supramolecular chemistry and nanoscience over recent years [4–5].

**Figure 1:**
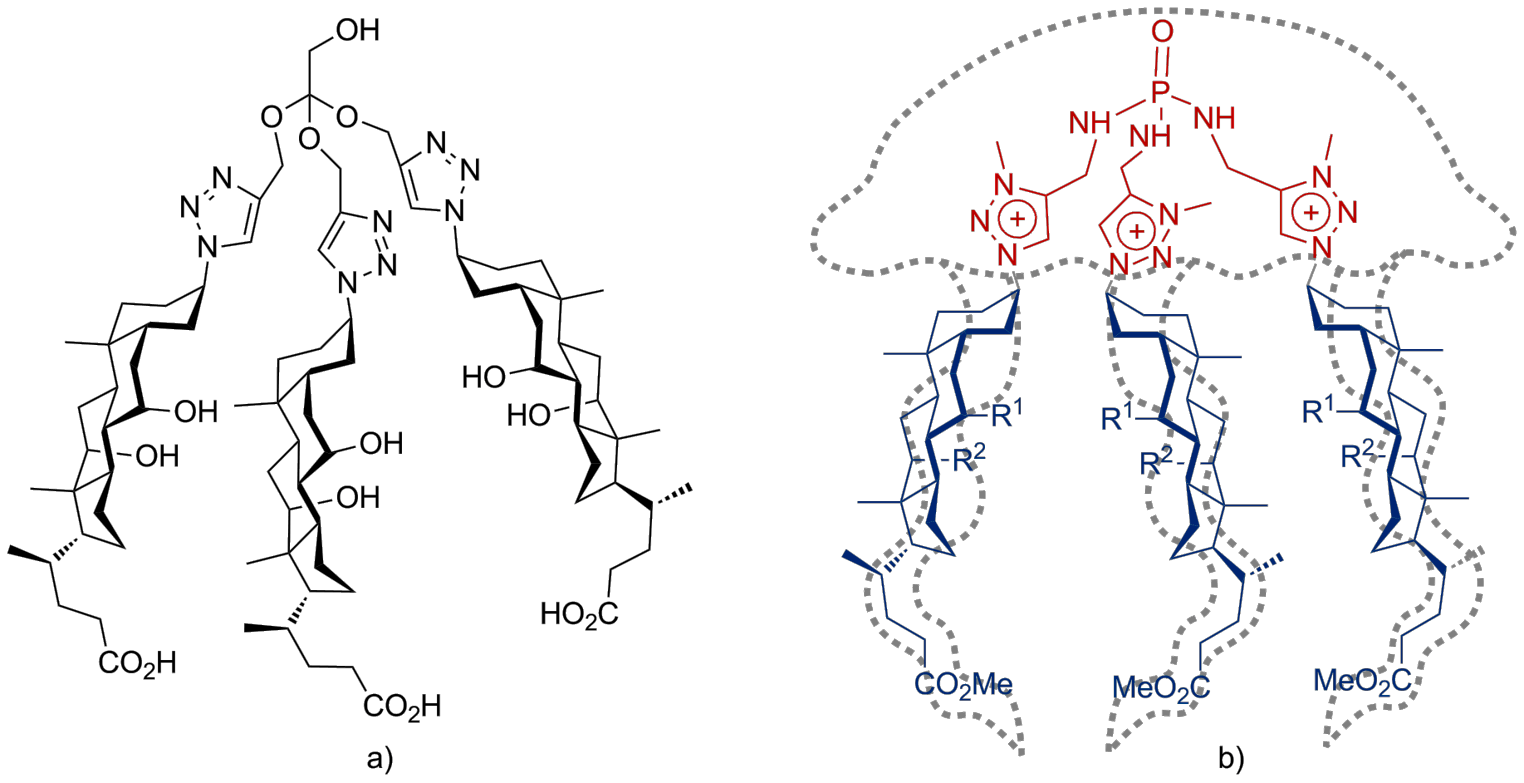
A tripodal molecular pocket (a) [12] or jellyfish resembling receptors (b) [11,16].

Many macrocyclic dimeric derivatives of bile acid were described [6–8]. As a rule, a route to the majority of macrocyclic structures requires multiple steps and is tedious, and the macrocyclization step generally provides low to moderate yields. Moreover, subsequent modification of these molecules for tuning their solubility or binding ability is often difficult. Consequently, the idea of molecular pockets or jellyfish resembling receptors with open “architecture” (Figure 1), which contain two or more bile acid fragments, has attracted more attention in recent years [9–16].

Bile acid, itself, can be used as a basis for ligand design [17–18]. Pincer-like ligands are capable of binding anions [18] or facilitate transport of anions through lipid membranes [19–22]. Some of similar pincer compounds can be used as receptors for organic molecules; e.g., polyamino derivatives based on cholane skeleton can form complexes with amino acids [23] and pincer-like conjugates of deoxycholic acid and aminopyrene can serve as sensors for aromatic compounds [24].

**Scheme 1:**
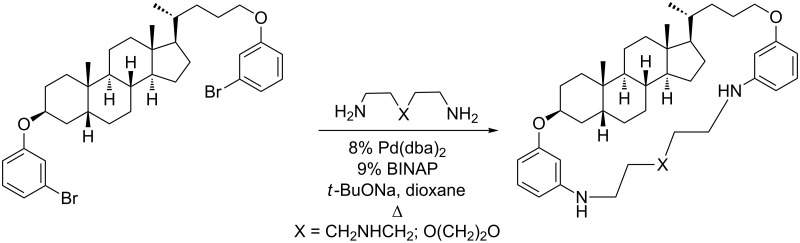
Example of Pd-catalyzed amination for modification of bile acid derivatives.

During the first decade of the XXI century, the main approaches to cyclic and pincer cholane derivatives were based on “classical” organic transformations (acylation, alkylation, etc.) [7–8,25–27]. Later, two modern synthetic approaches based on metal-catalyzed reactions were applied for the modification of bile acids – cross-coupling [28–29] (Scheme 1) and Cu(I)-catalyzed cycloaddition of azides to alkynes (Figure 1) [11,16,30–32].

We developed [33–35] a convenient technique for the synthesis of bile acids containing dimeric compounds and macrocycles by Pd-catalyzed Buchwald–Hartwig amination [36]. Though this method is often preferable for the macrocyclization in comparison to classical nucleophilic substitution [28,31], it is limited to bile acid derivatives bearing groups with C(sp^2^)–Hal bonds. In addition, the use of bile acid moieties saturated with hydroxy groups was inconvenient for the syntheses of bis(polyoxamino) derivatives by Pd-catalyzed amination [34–35].

Direct metal-catalyzed arylation of aminocholanes has not yet been employed in the synthesis of cholane-based ligands. Here, we report a new and efficient synthesis of arylaminocholanes and cholane-diaminoanthraquinone derivatives by Pd- and Cu-catalyzed amination. Such compounds not only have cation binding properties, but also might be easily introduced in lipid membranes to form ion channels. Appropriate close location of lipophilic steroid fragments in bis(cholanediamino)anthraquinones can be useful for non-covalent binding of lipids (such as cholesterol) or other lipophilic compounds.

## Results and Discussion

### Synthesis of bile acid derived ligands

**Scheme 2:**
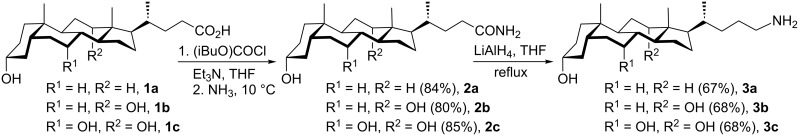
Synthesis of 24-aminocholanols.

A series of different 24-aminocholanols (compounds **3a–c**) was synthesized by reduction of the corresponding bile acid amides with LiAlH_4_ (Scheme 2) [37].

Cu-catalyzed amination is known to be a very efficient approach for C–N bond formation [38–39]. The availability of the variety of inexpensive ligands for copper(I) is a crucial benefit in comparison to the Pd-catalyzed variant. Primary amines of different structures can readily react with aryl halides to provide a wide range of products in excellent yields. However, yet there was no evidence of the same technique being applied to steroidal amines. Our particular interest was drawn by the synthesis of cholane-diaminoanthraquinone derivatives that can possess good complexation ability, chromophoric properties, and antitumor activity as well [40].

**Scheme 3:**
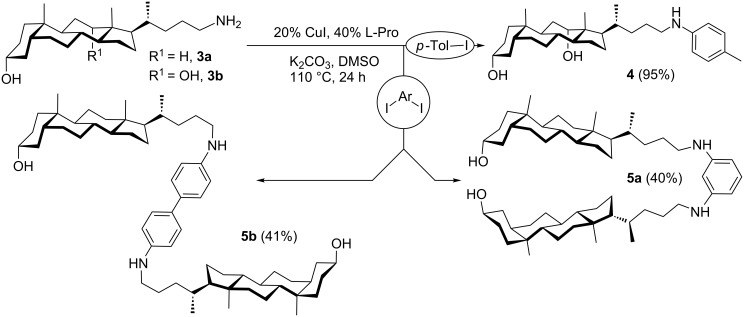
Synthesis of 24-arylaminocholanols by Cu-catalyzed amination.

We compared several CuI-based catalytic systems in the arylation of amine **3b** with 4-iodotoluene as a model substrate. We found out that the CuI/L-proline combination with K_2_CO_3_ led to the target aniline **4** in excellent yield (Scheme 3). Consequently, this system was used for the synthesis of bis-steroidal arylamines **5a** and **5b** starting from 24-aminocholanol **3a**.

Activated chlorinated substrates, such as 1,8- and 1,5-dichloroanthraquinones, are very promising substrates for nucleophilic substitution. For instance, 1,8-dichloro-9,10-anthraquinone (**6a**) is readily aminated by phthalimide using classical Ullmann conditions (15% Cu powder, quinoline, 200 °C in PhNO_2_) [41]. However, the reaction of **3c** with **6a** using the same conditions, as well as the addition of stronger bases (DABCO, DIPEA, DBU) afforded extremely low yields (7–12%) of aminated anthraquinones. Further attempts to apply the Cu-based catalytic system in this reaction with L-proline and other commonly used *N*,*N*, *N*,*O*, and *O*,*O* bidentate ligands were fruitless.

**Scheme 4:**
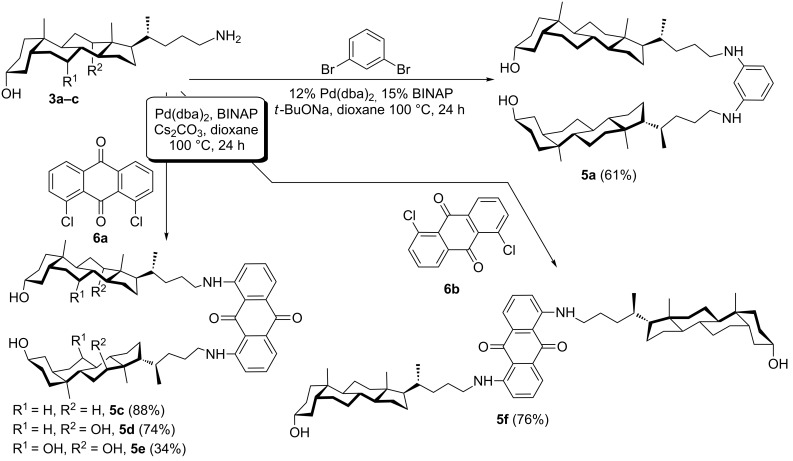
Synthesis of 24-arylaminocholanols by Pd-catalyzed amination.

Previously, some of us successfully used the classic conditions of the Buchwald–Hartwig amination for the preparation of bis-amino derivatives from various dibromo- and dichloroarenes [42]. Thus, we adopted the well performing Pd(dba)_2_/BINAP catalytic system for the reaction of aminocholanols **3a–c** with dibromo- and dichloroarenes (Scheme 4).

The synthesis of aniline **5a** was chosen to determine the viability of Pd-catalyzed amination. Though **5a** was not observed in the reaction with 1,3-diiodobenzene, a good yield was obtained with 1,3-dibromobenzene. In fact, the Pd-catalyzed coupling was more efficient [43] than Ullmann-type chemistry (61% from 1,3-dibromobenzene vs 40% from 1,3-diiodobenzene). Since the anthraquinone skeleton is rather sensitive to strong bases, we replaced NaO*t*-Bu with milder Cs_2_CO_3_ in reactions with dichlorides **6**. Both **6a** and **6b** gave good yields of aminoanthraquinones **5c** (88%) and **5f** (76%) under these conditions.

However, the arylation of deoxycholic acid derived aminocholane **3b** with **6a** gave a surprisingly low yield of **5d** (22%). We speculated that the low yield was caused by the presence of an additional hydroxy group in **3b**. The formation of the alkoxide anion on the substrate due to the thermodynamically unfavorable equilibrium with Cs_2_CO_3_ might promote the destruction of the anthraquinone scaffold. Indeed, the addition of an equimolar amount of iPrOH to the mixture of **3a** and **6a** under the same conditions led to the two-fold decrease in yield of **5c**. The replacement of BINAP with other phosphine ligands (*t*-Bu_3_P, dppf, PPF-NMe_2_) gave a very low turnover of Pd catalyst (up to 8 cycles).

The increase in reaction time (up to 65 h), reaction temperature (120 °C, *o*-xylene), and change of base to K_3_PO_4_ did not show any significant improvement in yield of **5d**. Fortunately, a high yield of **5d** (74%) was obtained by increasing the reagent concentrations to 0.25 M. The adjusted conditions were successfully applied in the arylation of cholic acid derived amine **3c** with **6a** affording 34% of **5e**. Thus, Pd-catalyzed amination seems to be a very competitive route for the preparation of arylated aminocholanes.

### Complexation of bile acid derived ligands

Steroidal ligands are known to be excellent hosts for different ions [8]. However, we did not observe complexation of pincer ligands **5c–e** with inorganic or organic anions by NMR titration. The attempt to increase the anion affinity for the ligands by exhaustive alkylation of amino groups with trimethyloxonium tetrafluorborate led exclusively to methylation of hydroxy groups.

**Figure 2:**
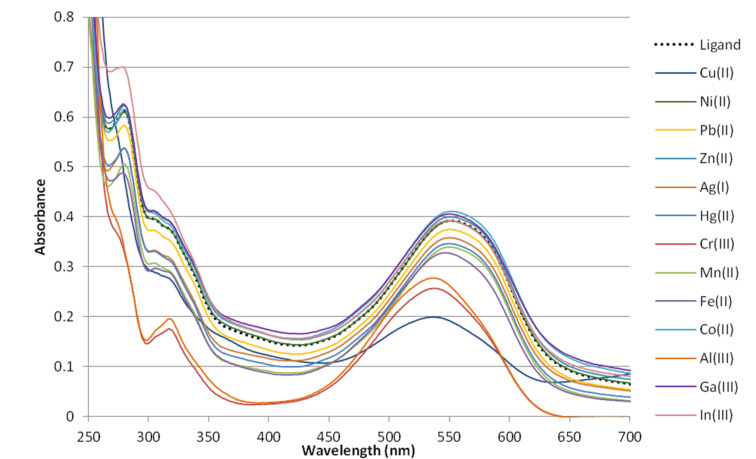
UV–vis spectra of **5c** (50 μM solution in MeCN) before and after the addition of 5 equiv of metal perchlorates.

In contrast the presence of the diaminoanthraquinone scaffold allows for easy detection of ion binding due to the high sensitivity of an intensive absorption band at 550 nm to the strength of intermolecular NH···O hydrogen bonds [44]. We studied the influence of the various cations (Al^3+^, Mn^2+^, Fe^2+^, Co^2+^, Ni^2+^, Zn^2+^, Cu^2+^, Ag^+^, Pb^2+^, Hg^2+^, Cr^3+^, Ga^3+^, Y^3+^, In^3+^) on the UV spectrum of **5c** (Figure 2). The qualitative selectivity test by stepwise addition of 1, 2, 3, and 5 equiv of metal perchlorates in MeCN to the solution of **5c** in MeCN revealed that the majority of cations did not influence the intensity of the absorption. However, addition of Al^3+^, Cr^3+^, and Cu^2+^ led to a decrease in absorption in the region of 500–600 nm and a hypsochromic shift of the maximum from 550 nm in a free ligand to 537 nm (Al^3+^), 539 nm (Cr^3+^), and 535 nm (Cu^2+^). To establish the stability and stoichiometry of complexes with these metals, the spectrophotometric titrations were carried out. Nonlinear regression analysis revealed the formation of the 1:1 complex of **5c** with Cu^2+^ and complexes with Al^3+^ and Cr^3+^ ions with different stoichiometry (Table 1). The same stoichiometry and binding constants were determined for deoxycholic acid derived ligand **5d**.

**Table 1:** Binding constants and stoichiometry of complexes of **5c** and **5d** with different cations.

Cation	Complex	log *K*	Complex	log *K*

Cu^2+^	(**5c**):Cu	4.40 ± 0.06	(**5d**):Cu	4.03 ± 0.07
Al^3+^	(**5c**)_2_:Al	8.25 ± 0.09	(**5d**)_2_:Al	9.22 ± 0.07
	(**5c**):Al	4.14 ± 0.07	(**5d**):Al	3.91 ± 0.07
Cr^3+^	(**5c**)_2_:Cr	8.07 ± 0.10	(**5d**)_2_:Cr	7.89 ± 0.06
	(**5c**):Cr	4.00 ± 0.08	(**5d**):Cr	3.85 ± 0.05

Thus, there is no appreciable dependence between the value of the binding constants and the presence of an extra hydroxy group in the cholane fragment. To the best of our knowledge a complex formation of 1,8-diaminoanthraquinone derivatives with Al and Cr salts have not been investigated earlier. The ligands derived of 1,8-diaminoanthraquinone are convenient for the detection of the copper cation [45–46]: the tetradentate ligand *N*,*N*’-bis(β-dimethylaminoethyl)-1,8-diaminoanthraquinone [47] demonstrate a two orders of magnitude better binding constant with copper cation in comparison to bidentate ligands **5c** and **5d**. However, the cholane skeleton in **5c** and **5d** can be useful for integration of these ligands into lipophilic membranes.

## Conclusion

Cu- and Pd-catalyzed cross-coupling reactions were applied for the preparation of arylamino derivatives of bile acids. High yields of arylaminocholanes were achieved in copper-catalyzed reactions of aryl iodides with 24-aminocholanes, while only a classical Pd-catalyzed protocol was suitable for the cross-coupling of aminocholanes and dichloroanthraquinones. The presence of hydroxy groups in the substrates was shown to decrease the yield of the Pd-catalyzed amination. This effect can be partially overridden by increasing concentrations of the reagents. The obtained bis(cholanylamino)anthraquinones demonstrated a high binding affinity to Cu^2+^, Al^3+^, and Cr^3+^.

## Supporting Information

Experimental procedures for compounds **2**–**5** and UV–vis titration data for **5c** and **5d**.

File 1 Experimental procedures, characterization data, copies of the ^1^H, ^13^C NMR spectra, UV–vis data.
