# Supplementary material for: Pd- and Cu-catalyzed approaches in the syntheses of new cholane aminoanthraquinone pincer-like ligands
Source: Beilstein J Org Chem. 2017 Mar 20;13:564–70. doi: 10.3762/bjoc.13.55 (PMC5372750; doi:10.3762/bjoc.13.55)
Supplement: File 1 — Experimental procedures, characterization data, copies of the 1H, 13C NMR spectra, UV–vis data. [file Beilstein_J_Org_Chem-13-564-s001.pdf]

# Supporting Information
# for
# Pd- and Cu-catalyzed approaches in the syntheses of new cholane aminoanthraquinone pincer-like ligands

Nikolay V. Lukashev*, Gennadii A. Grabovyi, Dmitry A. Erzunov, Alexey V. Kazantsev, Gennadij V. Latyshev, Alexei D. Averin and Irina P Beletskaya.

Address: Department of Chemistry, Lomonosov Moscow State University, Leninskie Gory 1-3, Moscow, 119991, Russia

Email: Nikolay V. Lukashev* - nvlukashev@gmail.com

*Corresponding author

## Experimental procedures, characterization data, copies of the $^{1}$H, $^{13}$C NMR spectra, UV–vis data

General information ........................................ S2

Experimental procedures and characterization data ........................................ S2

General procedure for preparation of amines 3a-c [2] ........................................ S3

General procedure of Cu-catalyzed amination ........................................ S5

General procedure of Pd-catalyzed amination ........................................ S6

Copies of the $^{1}$H and, $^{13}$C NMR spectra ........................................ S10

UV–vis titration ........................................ S26

References: ........................................ S33

# General information

$^{1}H$ and $^{13}C$-NMR spectra were recorded at 400 and 100.6 MHz respectively with a Bruker Avance 400 and Agilent MR 400 spectrometers. Chemical shifts were measured relative to HMDS and solvent signal in $^{1}H$ and $^{13}C$ NMR spectra respectively. Only characteristic signals in $^{1}H$-NMR of steroids are given. Column chromatography was carried out on Macherey-Nagel slilicagel 60 (0.040–0.063 mm). All procedures associated with oxygen- and water-sensitive compounds were conducted under dry argon atmosphere. $Cs_2CO_3$ was dried in vacuo at 120–150 °C for several hours, stored in a Schlenk tube, and weighed in an argon-flushed vessel.

UV–vis absorption spectra of a 50 µM solution of the ligands **5c** and **5d** in acetonitrile (3 ml placed into quartz cuvette with 1 cm optical path length) were recorded as a function of added $Cu(ClO_4)_2$, $Al(ClO_4)_3$ or $Cr(ClO_4)_3$ (0.01 M in acetonitrile) at a uniform data point interval of 1 nm with a Cary 60 (Agilent) spectrophotometer. All necessary aliquots of perchlorates (3.0 µl or 7.5 µl) were added manually with LLG-microliter pipette (0.5 to 10 µl). The calculation of stability constants was performed by nonlinear least-squares analysis with the Specfit program [1].

# Experimental procedures and characterization data

### General procedure for preparation of amides 2a–c [2]

To the suspension of bile acid (1.0 mmol) and $Et_3N$ (1.5 equiv) in THF (2.5 ml) isobutyl chloroformate (1.1 equiv) was added at 10 °C and reaction was stirred at this temperature for 15 min. Conc. aqueous $NH_3$ (1.5 equiv) was added dropwise and stirring was continued for 30 min at 10 °C and 3 h at rt. Solvents were evaporated and the crude product was washed with $H_2O$ followed by washing with $Et_2O$. The target product was dried in vacuo at 50–60 °C to constant mass and used without further purification.

**(3α,5β)-3-Hydroxycholan-24-amide (2a)**

Obtained from 1.50 g (4.0 mmol) of **1a**. White powder (84%, 1.26 g). M. p. 224-226 °C (lit. 214-216 °C [3], 208-210 °C [2]). $^{1}$H NMR (400 MHz, DMSO-$d_6$): 0.60 (3H, s, 18-$CH_3$), 0.86 (6H, 19-$CH_3$, 21-$CH_3$), 2.01 (2H, m, 23-$CH_2$), 3.35 (1H, m, 3β-H), 4.43 (1H, d, *J* 4.5 Hz, 3-OH), 6.63 (1H, s, C(O)$NH_2$), 7.20 (1H, s, C(O)$NH_2$).

**(3α,5β,12α)-3,12-Dihydroxycholan-24-amide (2b)**

Obtained from 5.0 g (12.7 mmol) of **1b**. White powder (80%, 4.0 g). M. p. 213-215 °C (lit. 213-215 °C [2]). $^{1}$H NMR (400 MHz, DMSO-$d_6$): 0.58 (3H, s, 18-$CH_3$), 0.83 (3H, s, 19-$CH_3$), 0.90 (3H, d, *J* 6.4 Hz, 21-$CH_3$), 1.98 (2H, m, 23-$CH_2$), 3.36 (1H, m, 3β-H), 3.59 (1H, m, 12β-H), 4.17 (1H, s, 12-OH), 4.45 (1H, s, 3-OH), 6.61 (1H, s, C(O)$NH_2$), 7.19 (1H, s, C(O)$NH_2$).

**(3α,5β,7α,12α)-3,7,12-Trihydroxycholan-24-amide (2c)**

Obtained from 5.0 g (12.2 mmol) of **1c**. White powder (85%, 4.3 g). M. p. 138-140°C (lit. 135-137°C [2]). $^{1}$H NMR (400 MHz, DMSO-$d_6$): 0.57 (3H, s, 18-$CH_3$), 0.80 (3H, s, 19-$CH_3$), 0.91 (3H, d, *J* 5.4 Hz, 21-$CH_3$), 2.15 (2H, m, 23-$CH_2$), 3.17 (1H, m, 3β-H), 3.60 (1H, br.s, 12β-H), 3.77 (1H, br. s, 7β-H), 4.01 (1H, s, 7-OH), 4.10 (1H, s, 12-OH), 4.32 (1H, s, 3-OH), 6.63 (1H, s, C(O)$NH_2$), 7.21 (1H, s, C(O)$NH_2$).

**General procedure for preparation of amines 3a–c [2]**

To the cooled (with an ice bath) suspension of amide **2** (1.0 mmol) in THF (10 ml) $LiAlH_4$ (3 equiv) was added in small portions (**CAUTION**: exothermic reaction with hydrogen evolution) under argon atmosphere. When evolution of hydrogen ceased the mixture was refluxed for 15 h, cooled to rt, and excess of $LiAlH_4$ was quenched by dropwise addition of $H_2O$ (3 ml). The suspension was half

concentrated and then filtered. The white solid was washed with hot THF until disappearance of the amine **3** in the filtrate (TLC control). The filtrate was evaporated and the product was purified by column chromatography.

**(3α,5β)-3-Hydroxycholan-24-amine (3a)**

Obtained from 1.26 g (3.4 mmol) of **2a**. Eluent $CH_2Cl_2$:$CH_3OH$:$Et_3N$ 100:10:5, $R_f$ 0.5. White powder (67%, 0.83 g). M. p. 158-160 °C (lit. 148-150 °C [2]). $^1$H NMR (400 MHz, DMSO-$d_6$): 0.63 (3H, s, 18-$CH_3$), 0.90 (6H, s, 19-$CH_3$, 21-$CH_3$), 1.95 (2H, m, 24-$CH_2$), 2.64 (2H, m, $NH_2$), 3.58 (1H, m, 3β-H).

**(3α,5β,12α)-3,12-Dihydroxycholan-24-amine (3b)**

Obtained from 4.0 g (10.2 mmol) of **2b**. Eluent $CH_2Cl_2$:$CH_3OH$:$Et_3N$ 100:10:5, $R_f$ 0.3. White powder (68%, 2.6 g). M. p. 108-110°C. $^1$H NMR (400 MHz, DMSO-$d_6$): 0.58 (3H, s, 18-$CH_3$), 0.83 (3H, s, 19-$CH_3$), 0.91 (3H, d, *J* 6.5 Hz, 21-$CH_3$), 2.45 (2H, m, $NH_2$), 3.35 (1H, m, 3β-H), 3.78 (1H, s, 12β-H). $^{13}$C NMR (100.6 MHz, DMSO-$d_6$): 11.8, 12.5, 17.4, 23.1, 23.5, 26.1, 27.0, 27.4, 28.7, 30.1, 30.2, 32.9, 34.4, 34.9, 35.3, 36.3, 41.6, 42.3, 45.7, 45,9, 46.3, 47.5, 69.9, 71.0.

**(3α,5β,7α,12α)-3,7,12-Trihydroxycholan-24-amine (3c)**

Obtained from 4.3 g (10.4 mmol) of **2c**. Eluent $CH_2Cl_2$:$CH_3OH$:$Et_3N$ 100:10:5, $R_f$ 0.2. White powder (68%, 2.8 g, 7.1 mmol). M. p. 110-112°C. $^1$H NMR (400 MHz, DMSO-$d_6$): 0.58 (3H, s, 18-$CH_3$), 0.80 (3H, s, 19-$CH_3$), 0.91 (3H, d, *J* 7.0 Hz, 21-$CH_3$), 3.17 (1H, m, 3β-H), 3.60 (1H, s, 12β-H), 3.78 (1H, s, 7β-H), 4.00 (1H, s, 7-OH), 4.09 (1H, s, 12-OH), 4.30 (1H, br. s, 3-OH). $^{13}$C NMR (100.6 MHz, DMSO-$d_6$): 11.8, 12.4, 17.4, 23.1, 23.5, 26.1, 27.0, 27.3, 28.6, 30.1, 30.2, 32.9, 33.8, 35.1, 35.3, 35.4, 41.3, 41.5, 42.3, 45.7, 46.2, 66.2, 70.3, 71.0.

**General procedure of Cu-catalyzed amination**

A mixture of amine **3** (0.3 mmol), aryl halide (0.1 mmol), CuI (0.02 mmol), L-proline (0.04 mmol) and $K_2CO_3$ (0.4 mmol) in DMSO (1 ml) was stirred at 110 °C in a glass vial with a screw cap under argon atmosphere for 24 h. The reaction mixture was cooled to rt, diluted with $CH_2Cl_2$ (15 ml) and washed with $H_2O$ (3 × 10 ml). The organic layer was dried over $Na_2SO_4$, evaporated, and the product was purified by column chromatography.

**4-[(3α,5β,12α)-3,12-Dihydroxycholan-24-ylamino]toluene (4)**

Obtained from 56.7 mg (0.15 mmol) of **3b**, 21.8 mg (0.1 mmol) of 4-iodotoluene, 3.8 mg (0.02 mmol) of CuI, 4.6 mg (0.04 mmol) of L-proline, 27.6 mg (0.2 mmol) of $K_2CO_3$ in DMSO (1 ml). Pale-brown powder (95%, 44.4 mg). $^1$H NMR (400 MHz, DMSO-$d_6$): 0.59 (3H, s, 18-$CH_3$), 0.83 (3H, s, 19-$CH_3$), 0.93 (3H, d, *J* 6.5 Hz, 21-$CH_3$), 2.12 (3H, s, $CH_3$), 3.78 (1H, s, 12β-H), 4.19 (1H, d, *J* 3.8 Hz, 12-OH), 4.47 (1H, d, *J* 3.8 Hz , 3-OH), 5.22 (1H, t, *J* 5.6 Hz., NH), 6.43 (2H, d, *J* 8.2 Hz), 6.85 (2H, d, *J* 8.2 Hz). $^{13}$C NMR (100.6 MHz, $CDCl_3$): 12.7, 17.6, 20.3, 23.1, 23.6, 26.1, 26.3, 27.1, 27.6, 28.5, 30.4, 33.2, 33.6, 34.1, 35.2, 35.4, 36.0, 36.4, 42.0, 44.9, 46.4, 47.5, 48.2, 71.7, 73.14, 112.9, 126.3, 129.6, 146.2

**1,3-Bis[(3α,5β)-3-hydroxycholan-24-ylamino]benzene (5a)**

Pale-brown powder (40%, 31.9 mg). M.p. 123-125 $^o$C. $^1$H NMR (400 MHz, $CDCl_3$): 0.63 (6H, s, 18-$CH_3$), 0.91 (6H, s, 19-$CH_3$), 0.92 (6H, d, *J* 6.4 Hz, 21-$CH_3$), 3.03 (4H, m, 24-$CH_2$), 3.61 (2H, m, 3β-H), 5.85 (1H, t, *J* 1.9 Hz, 2-$H_{Ar}$), 5.98 (2H, dd, *J* 8.0 Hz, 1.9 Hz, 4-$H_{Ar}$ and 6-$H_{Ar}$), 6.95 (1H, t, *J* 8.0 Hz, 5-$H_{Ar}$). $^{13}$C NMR (100.6 MHz, $CDCl_3$): 12.0, 18.6, 20.8, 23.3, 24.2, 26.2, 26.4, 27.2, 28.3, 30.5, 33.3, 34.5, 35.3, 35.6, 35.8, 36.4, 40.1, 40.4, 42.0, 42.7, 44.6, 56.1, 56.4, 71.8, 76.7, 77.0, 77.3, 96.9, 102.7, 129.8, 149.7.

Calculated ($C_{54}H_{88}N_2O_2$): C 81.35%, H 11.13%, N 3.51%.Found: C 81.47%, H 11.12%, N 3.55%.

**4,4'-Bis[(3α,5β)-3-hydroxycholan-24-ylamino]biphenyl (5b)**

White powder (41%, 35.8 mg). M.p. 133-135 °C. $^1$H NMR (400 MHz, $CDCl_3$): 0.64 (6H, s, 18-$CH_3$), 0.91 (6H, s, 19-$CH_3$), 0.92 (6H, d, *J* 6.5 Hz, 21-$CH_3$), 3.08 (4H, m., 24-$CH_2$), 3.61 (2H, m, 3β-H), 6.64 (4H, d, *J* 7.6 Hz, *m*-$H_{Ar}$), 7.35 (4H, d, *J* 7.6 Hz, *o*-$H_{Ar}$). $^{13}$C NMR (100.6 MHz, $CDCl_3$): 12.0, 18.7, 20.8, 23.3, 24.2, 26.2, 26.4, 27.2, 28.3, 30.5, 33.3, 34.6, 35.3, 35.6, 35.8, 36.5, 40.2, 40.4, 42.1, 42.7, 44.8, 56.2, 56.5, 71.9, 113.1, 127.1, 130.6, 146.9.

Calculated ($C_{60}H_{92}N_2O_2 \cdot CH_2Cl_2$): C 76.45%, H 9.89%, N 2.92%. Found: C 76.22%, H 9.60%, N 3.11 %.

**General procedure of Pd-catalyzed amination**

A mixture of amine **3** (0.3 mmol), aryl halide (0.1 mmol), Pd(dba)$_2$ (0.012 mmol), BINAP (0.015 mmol) and $Cs_2CO_3$ (0.4 mmol) in dioxane (2 ml) was stirred at 100 °C in a glass vial with a screw cap under argon atmosphere for 24 h. The reaction mixture was cooled to rt, diluted with $CH_2Cl_2$ (15 ml) and washed with $H_2O$ (3 × 10 ml). The organic layer was dried over $Na_2SO_4$, evaporated, and the product was purified by column chromatography.

**1,3-bis[(3α,5β)-3-hydroxycholan-24-ylamino]benzene (5a)**

Obtained from 65.1 mg (0.18 mmol) of **3a**, 7.1 μl (0.06 mmol) of 1,3-dibromobenzene, 4.1 mg (0.0072 mmol) of Pd(dba)$_2$, 5.6 mg (0.009 mmol) of BINAP, 34.6 mg (0.36 mmol) of *t*-BuONa in dioxane (1 ml). Pale-brown powder (61%, 29.2 mg).

**1,8-Bis[(3α,5β)-3-hydroxycholan-24-ylamino]-9,10-anthraquinone (5c)**

Obtained from 108.5 mg (0.3 mmol) of **3a** and 27.7 mg (0.1 mmol) of 1,8-dichloro-9,10-anthraquinone. Dark-violet powder (88%, 81,6 mg). M.p. 156-158 $^{o}$C. $^{1}$H NMR (400 MHz, $CDCl_3$): 0.62 (6H, s, 18-$CH_3$), 0.90 (6H, s, 19-$CH_3$), 0.94 (6H, d, *J* 6.5 Hz, 21-$CH_3$), 3.25 (4H, m, 24-$CH_2$), 3.61 (2H, m, 3β-H), 6.99 (2H, d, *J* 8.4 Hz, *o*-$H_{Ar}$), 7.45 (2H, t, *J* 8.4 Hz, *m*-$H_{Ar}$), 7.52 (2H, d, *J* 7.3 Hz, *p*-$H_{Ar}$), 9.66 (2H, t, *J* 4.9 Hz, NH). $^{13}$C NMR (100.6 MHz, $CDCl_3$): 12.1, 18.7, 20.8, 23.4, 24.3, 25.8, 26.5, 27.2, 28.3, 30.6, 33.5, 34.6, 35.4, 35.7, 35.9, 36.5, 40.2, 40.4, 42.1, 42.7, 43.6, 56.1, 56.5, 71.8, 114.3, 114.8, 117.7, 134.1, 134.4, 151.2, 184.8, 188.9.

Calculated ($C_{62}H_{90}N_2O_4$): C 80.30%, H 9.78%, N 3.02%. Found: C 80.63%, H 9.60%, N 2.91 %.

**1,8-bis[(3α,5β,12α)-3,12-dihydroxycholan-24-ylamino]-9,10-anthraquinone (5d)**

Obtained from 108.5 mg (0.3 mmol) of **3b**, 27.7 mg (0.1 mmol) of **6a**, 2.3 mg (0.004 mmol) of Pd(dba)$_2$, 3.1 mg (0.005 mmol) of BINAP, 130.3 mg (0.4 mmol) of $Cs_2CO_3$ and 0.4 ml of dioxane. Dark-violet powder (74%, 71 mg). M.p. 180-182 $^{o}$C. $^{1}$H NMR (400 MHz, $CDCl_3$): 0.67 (6H, s, 18-$CH_3$), 0.89 (6H, s, 19-$CH_3$), 1.03 (6H, d, *J* 6.4 Hz, 21-$CH_3$), 3.24 (4H, m, 24-$CH_2$), 3.59 (2H, m, 3β-H), 3.99 (2H, br. s., 12β-H), 6.98 (2H, d, *J* 8.4 Hz, *o*-$H_{Ar}$), 7.44 (2H, t, *J* 8.3 Hz, *m*-$H_{Ar}$) 7.50 (2H, d, *J* 7.3 Hz, *p*-$H_{Ar}$), 9.61 (2H, br. s., NH). $^{13}$C NMR (100.6 MHz, $CDCl_3$): 12.7, 17.8, 23.1, 23.7, 25.7, 26.1, 27.1, 27.6, 28.6, 30.5, 33.5, 33.6, 34.1, 35.2, 35.4, 36.0, 36.4, 42.0, 43.6, 46.5, 47.4, 48.3, 71.7, 73.2, 114.3, 114.7, 117.7, 134.0, 134.3, 151.2, 184.7, 188.9.

Calculated ($C_{62}H_{90}N_2O_6 \cdot 3CH_2Cl_2$): C 65.30%, H 7.97%, N 2.31%. Found: C 65.68%, H 7.88%, N 2.27%.

**1,8-bis[(3α,5β,12α)-3,7,12-Trihydroxycholan-24-ylamino]-9,10-anthraquinone (5e)**

Obtained from 118.1 mg (0.3 mmol) of **3c**, 27.7 mg (0.1 mmol) of **6a**, 2.3 mg (0.004 mmol) of $Pd(dba)_2$, 3.1 mg (0.005 mmol) of BINAP, 130.3 mg (0.4 mmol) of $Cs_2CO_3$ and 0.4 ml of dioxane. Violet powder (34%, 33.7 mg). M.p. 171-173 $^{o}C$. $^{1}H$ NMR (400 MHz, $CDCl_3$): 0.63 (6H, s, 18-$CH_3$), 0.82 (6H, s, 19-$CH_3$), 1.02 (6H, d, *J* 6.4 Hz, 21-$CH_3$), 3.25 (2H, m, 3β-H), 3.35 (4H, m, 24-$CH_2$), 3.8 (2H, br. s., 7β-H), 3.96 (2H, br. s., 12β-H), 7.01 (2H, d, *J* 8.4 Hz, *o*-$H_{Ar}$), 7.44 (2H, t, *J* 8.3 Hz, *m*-$H_{Ar}$) 7.50 (2H, d, *J* 7.3 Hz, *p*-$H_{Ar}$), 9.70 (2 H, br. s., NH). $^{13}C$ NMR (100.6 MHz, $CDCl_3$): 12.3, 17.8, 22.4, 23.3, 25.1, 26.3, 28.0, 28.1, 30.5, 33.6, 34.7, 34.9, 35.3, 35.6, 39.3, 41.5, 41.70, 43.2, 46.4, 47.2, 68.5, 72.1, 73.3, 114.5, 114.9, 117.8, 134.1, 134.4, 151.1, 184.6, 188.8.

Calculated ($C_{62}H_{90}N_2O_8 \cdot 3CH_3OH$): C 71.79%, H 9.45%, N 2.58%. Found: C 71.93%, H 9.41%, N 2.45%.

**1,5-bis[(3α,5β)-3-Hydroxycholan-24-ylamino]-9,10-anthraquinone (5f)**

Obtained from 108.5 mg (0.3 mmol) of **3a** and 27.7 mg (0.1 mmol) **6b**. Dark-violet powder (76%, 70,5 mg). M.p. 152-154 $^{o}C$. $^{1}H$ NMR (400 MHz, $CDCl_3$): 0.64 (6H, s, 18-$CH_3$), 0.91 (6H, s, 19-$CH_3$), 0.95 (6H, d, *J* 6.4 Hz, 21-$CH_3$), 3.26 (4H, m, 24-$CH_2$), 3.61 (2H, m, 3β-H), 6.94 (2H, d, *J* 8.1 Hz, *o*-$H_{Ar}$), 7.51 (4H, m, *m*-$H_{Ar}$ and *p*-$H_{Ar}$), 9.69 (2H, t, *J* 4.9 Hz, NH). $^{13}C$ NMR (100.6 MHz, $CDCl_3$): 12.0, 18.6, 20.8, 23.4, 24.2, 25.8, 26.4, 27.2, 28.3, 30.5, 33.4, 34.5, 35.3, 35.6, 35.8, 36.4, 40.1, 40.4, 42.1, 42.7, 43.5, 56.0, 56.5, 71.8, 112.8, 114.6, 116.3, 135.1, 136.3, 151.4, 185.4.

Calculated ($C_{62}H_{90}N_2O_4$): C 80.30%, H 9.78%, N 3.02%. Found: C 79.97%, H 9.57%, N 2.78 %.

**1,8-Bis[(3α,5β)-3-methoxycholan-24-ylamino]-9,10-anthraquinone (S1)**

A mixture of **5c** (46 mg, 0.05 mmol) and trimethyloxonium tetrafluoroborate (17.7 mg, 0.12 mmol) in dry $CH_2Cl_2$ (5 ml) was stirred at ambient temperature under argon atmosphere for 24 h. The

reaction mixture was diluted with $CH_2Cl_2$ (15 ml) and washed with $H_2O$ (10 ml). The organic layer was dried over $Na_2SO_4$, evaporated, and the product was purified by column chromatography. Dark-violet powder (29%, 14 mg). $^1$H NMR (400 MHz, $CDCl_3$): 0.66 (6H, s, 18-$CH_3$), 0.92 (6H, s, 19-$CH_3$), 0.98 (6H, d, *J* 6.5 Hz, 21-$CH_3$), 3.16 (2H, m, 3β-H), 3.26 (4H, m, 24-$CH_2$), 3.35 (6H, s, 3-$OCH_3$), 7.00 (2H, d, *J* 8.1 Hz, *o*-$H_{Ar}$), 7.46 (2H, t, *J* 8.1 Hz, *m*-$H_{Ar}$) 7.52 (2H, d, *J* 7.3 Hz, *p*-$H_{Ar}$), 9.63 (2H, t, J 4.9 Hz., NH). $^{13}$C NMR (100.6 MHz, $CDCl_3$): 12.1, 18.7, 20.8, 23.4, 24.3, 25.8, 26.4, 27.3, 28.3, 29.7, 32.7, 33.4, 34.9, 35.3, 35.6, 35.9, 40.2, 40.3, 42.0, 42.7, 43.5, 55.5, 56.1, 56.5, 80.4, 114.3, 114.7, 117.6, 134.0, 134.3, 151.2, 184.8, 188.9.

## Copies of the $^{1}$H and $^{13}$C NMR spectra

$^{1}$H NMR spectrum of 4-[(3α,5β,12α)-3,12-dihydroxycholan-24-ylamino]toluene (**4**) (DMSO-$d_6$, 400 MHz, 300 K)

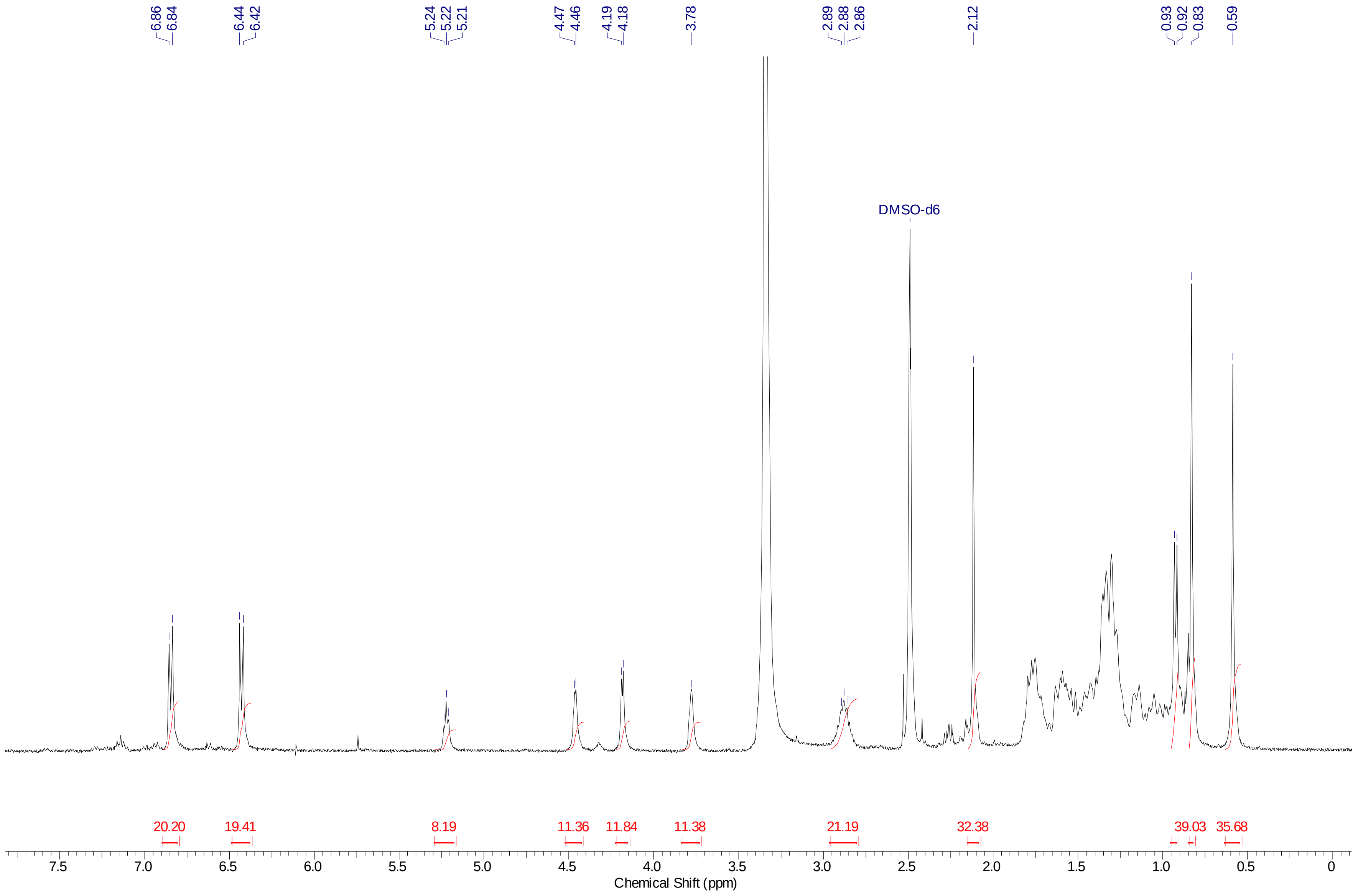

$^{13}C$ NMR spectrum of 4-[(3α,5β,12α)-3,12-dihydroxycholan-24-ylamino]toluene (**4**) ($CDCl_3$, 100.6 MHz, 300 K)

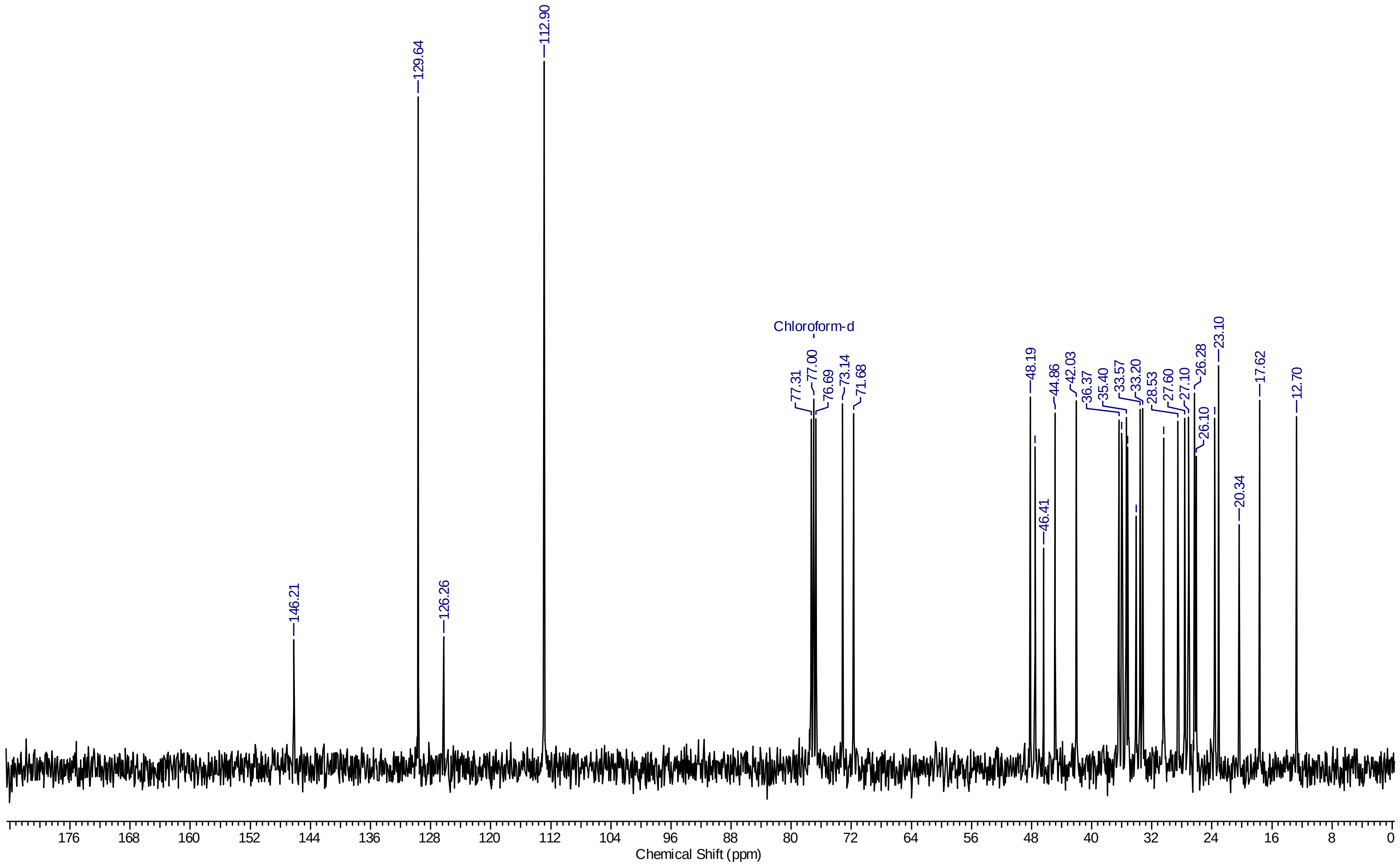

$^{1}$H NMR spectrum of 1,3-bis[(3α,5β)-3-hydroxycholan-24-ylamino]benzene (**5a**) ($CDCl_3$, 400 MHz, 300 K)

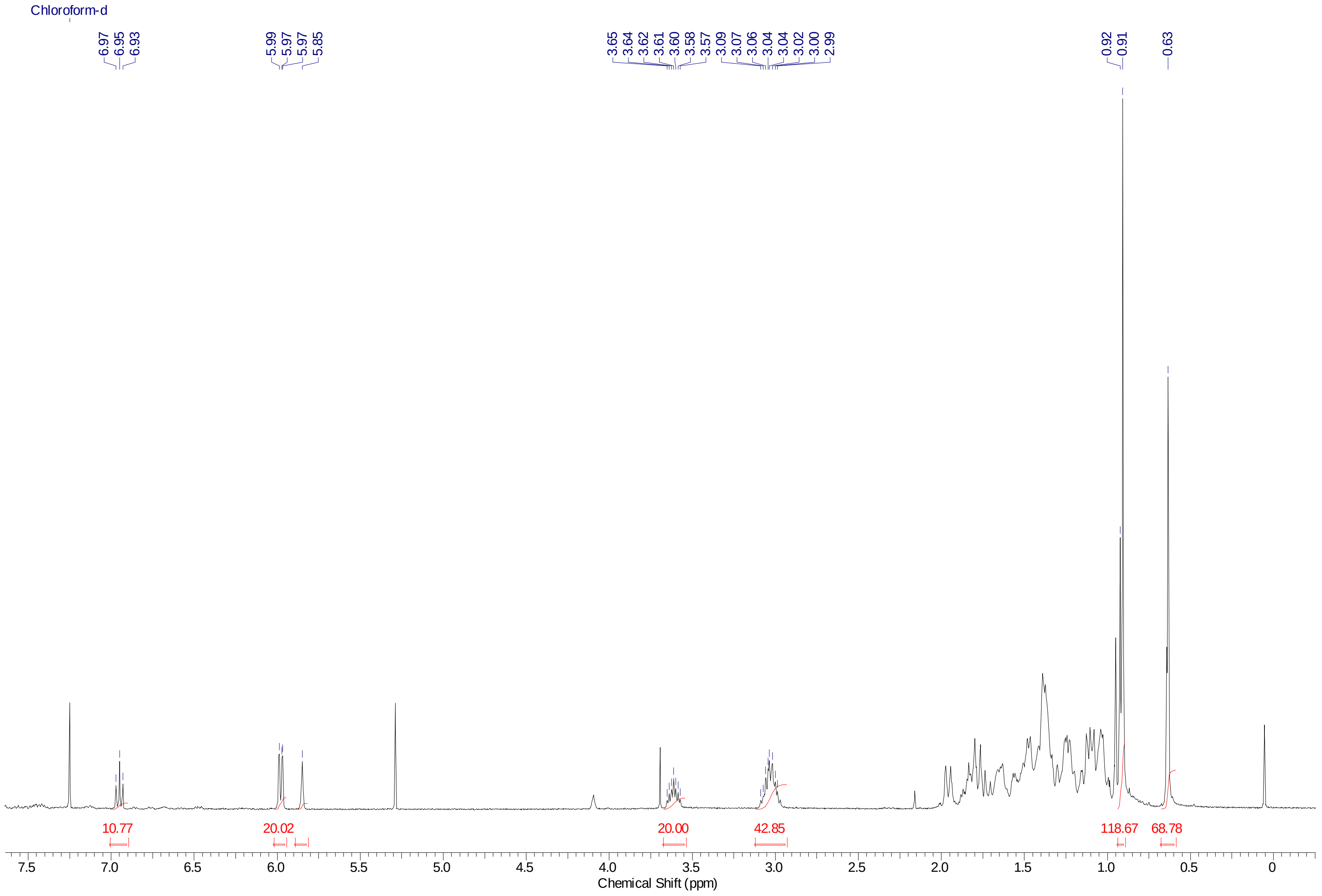

$^{13}$C NMR spectrum of 1,3-bis[(3α,5β)-3-hydroxycholan-24-ylamino]benzene (**5a**) ($CDCl_3$, 100.6 MHz, 300 K)

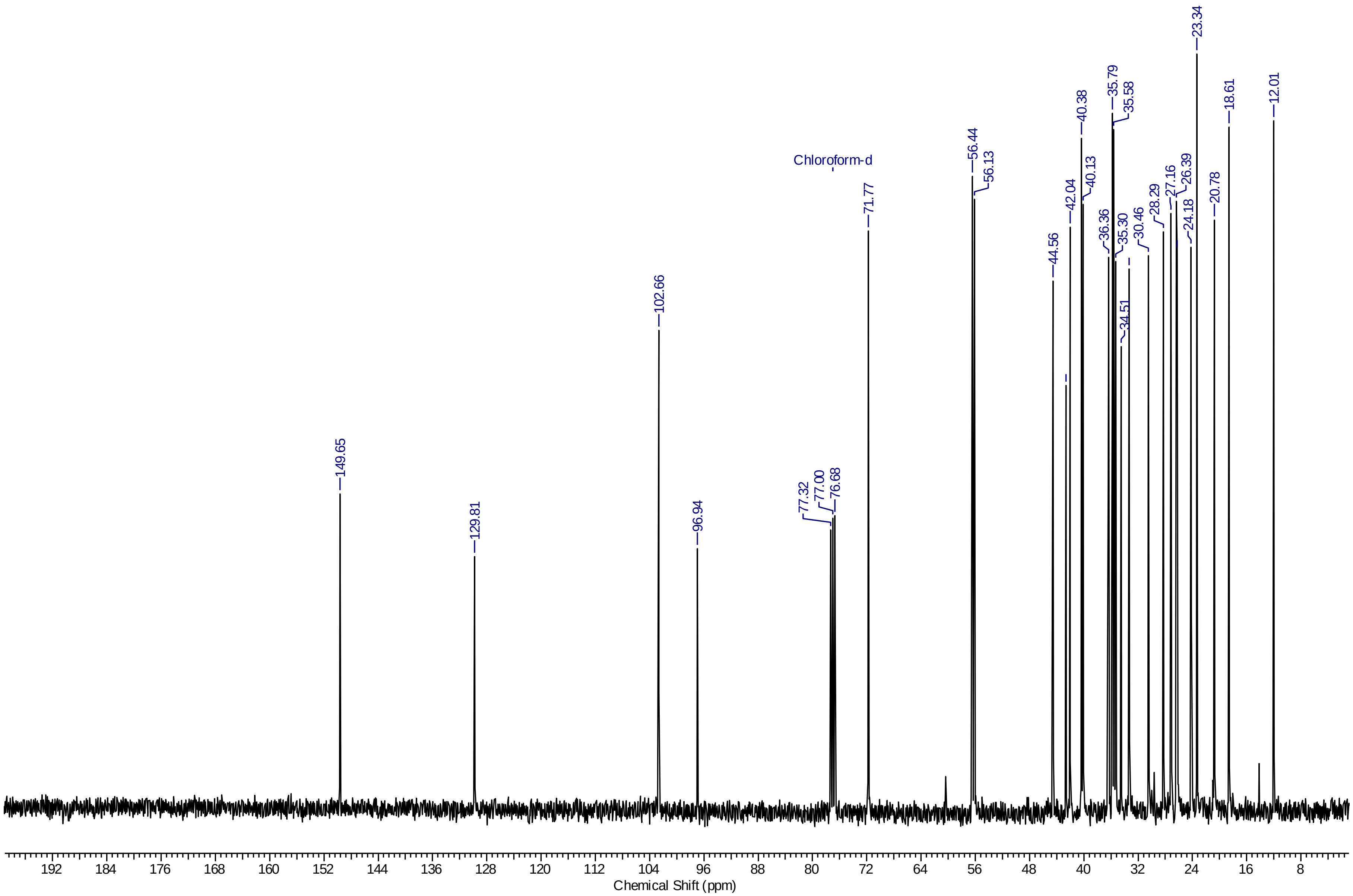

$^{1}$H NMR spectrum of 4,4'-bis[(3α,5β)-3-hydroxycholan-24-ylamino]biphenyl (**5b**) ($CDCl_3$, 400 MHz, 300 K)

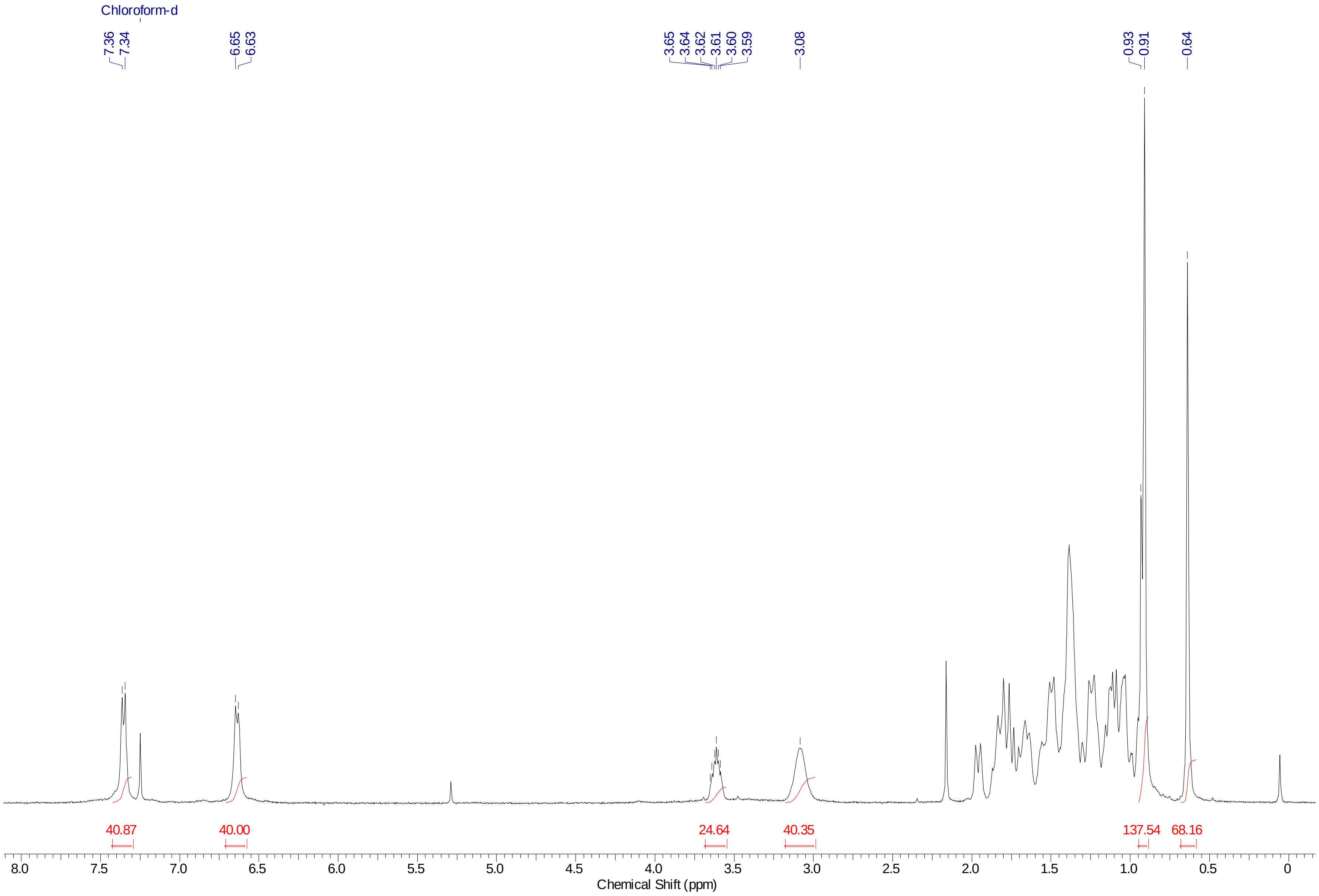

$^{13}C$ NMR spectrum of 4,4'-bis[(3α,5β)-3-hydroxycholan-24-ylamino]biphenyl (**5b**) ($CDCl_3$, 100.6 MHz, 300 K)

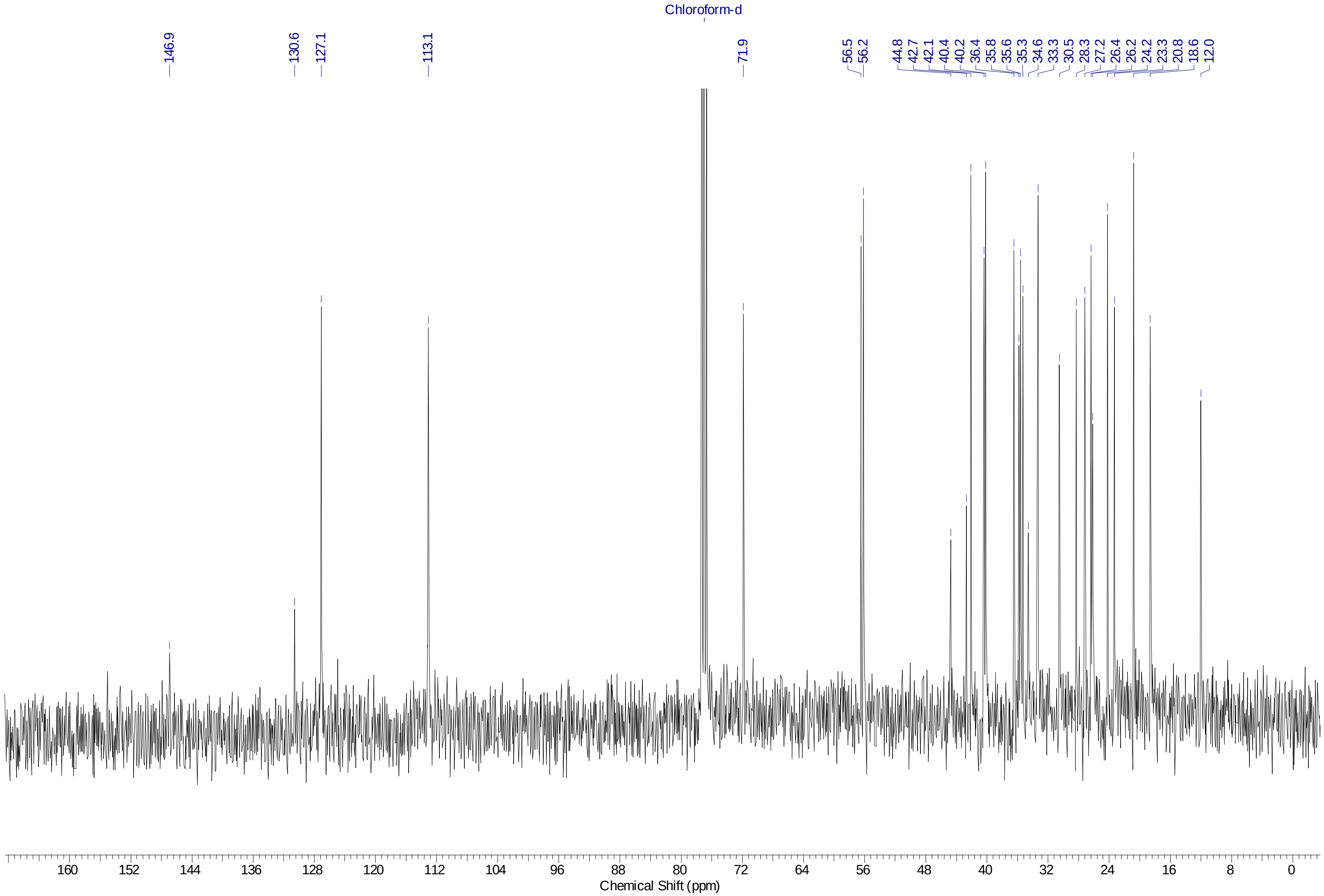

$^{1}$H NMR spectrum of 1,8-bis[(3α,5β)-3-hydroxycholan-24-ylamino]-9,10-anthraquinone (**5c**) ($CDCl_3$, 400 MHz, 300 K)

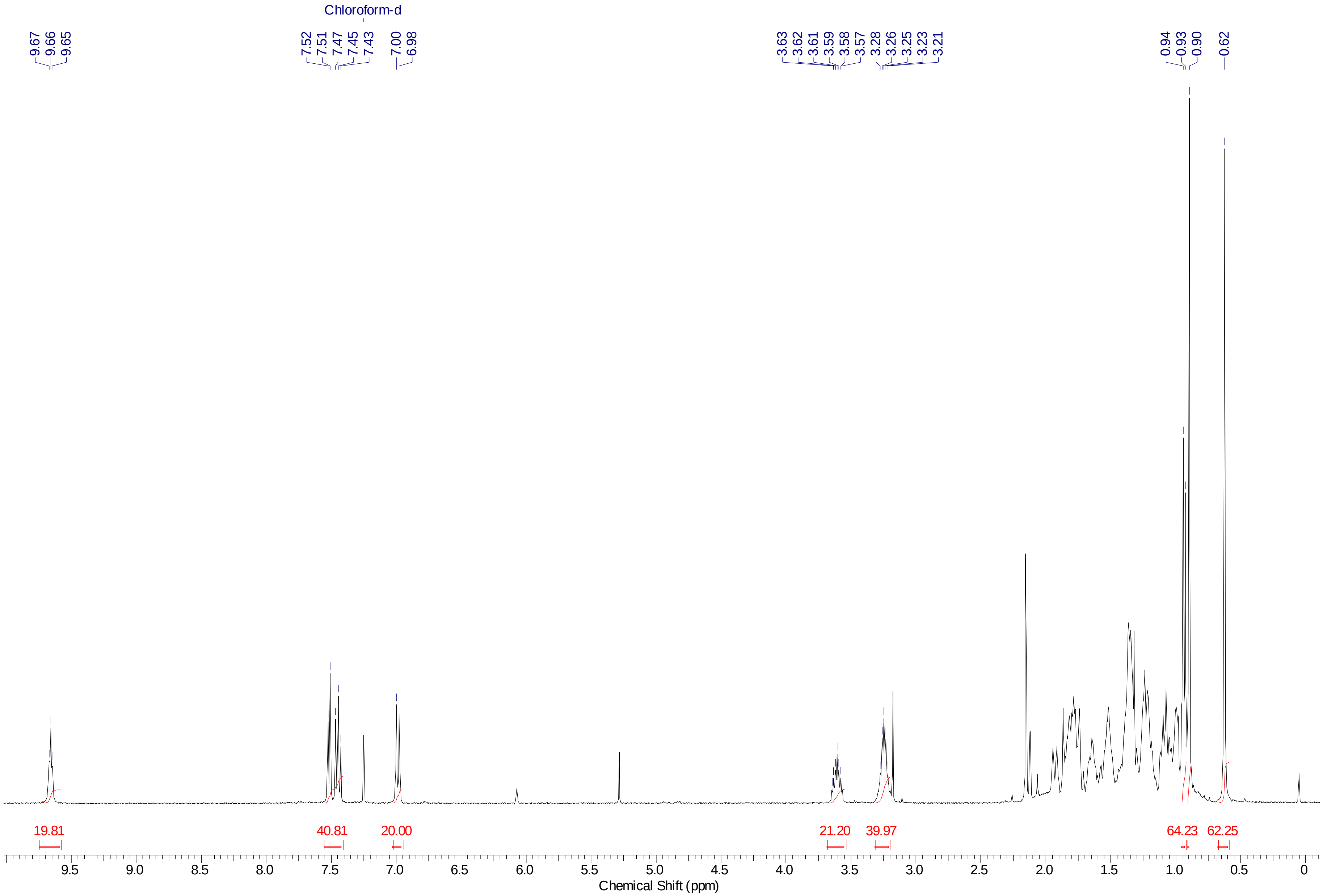

$^{13}$C NMR spectrum of 1,8-bis[(3α,5β)-3-hydroxycholan-24-ylamino]-9,10-anthraquinone (**5c**) ($CDCl_3$, 100.6 MHz, 300 K)

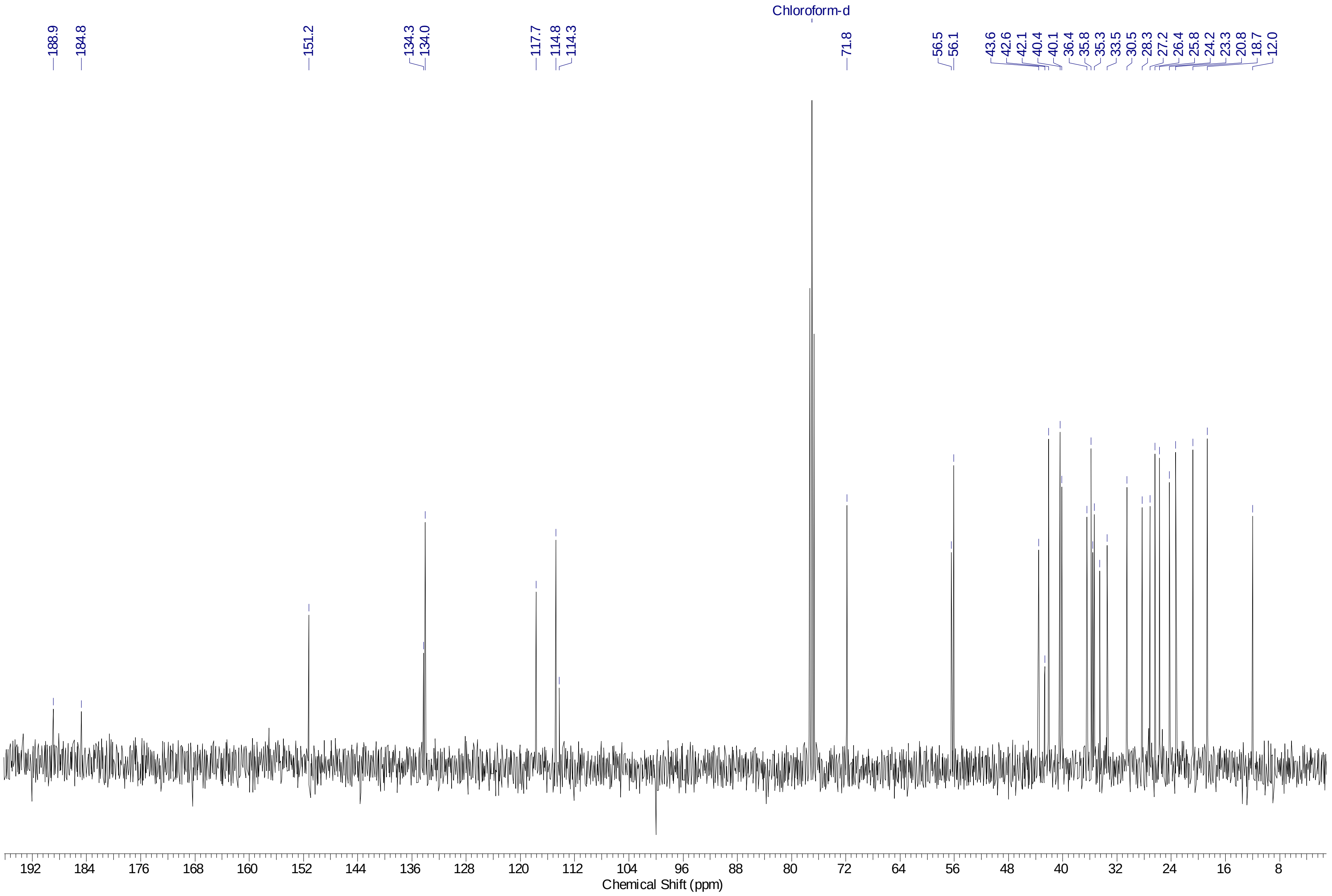

$^{1}$H NMR spectrum of 1,8-bis[(3α,5β,12α)-3,12-dihydroxycholan-24-ylamino]-9,10-anthraquinone (**5d**) (CDCl$_3$, 400 MHz, 300 K)

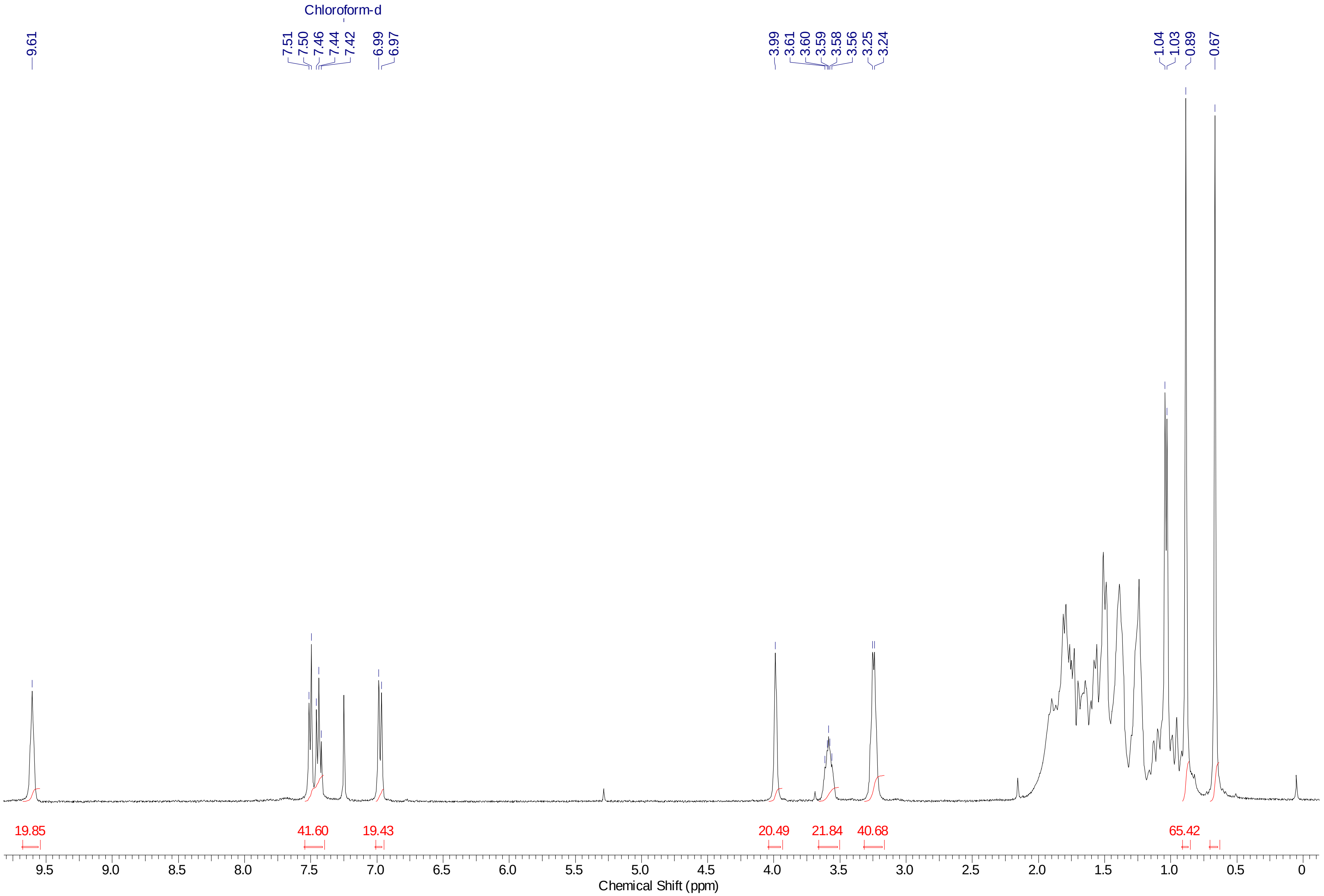

$^{13}$C NMR spectrum of 1,8-bis[(3α,5β,12α)-3,12-dihydroxycholan-24-ylamino]-9,10-anthraquinone (**5d**) ($CDCl_3$, 100.6 MHz, 300 K)

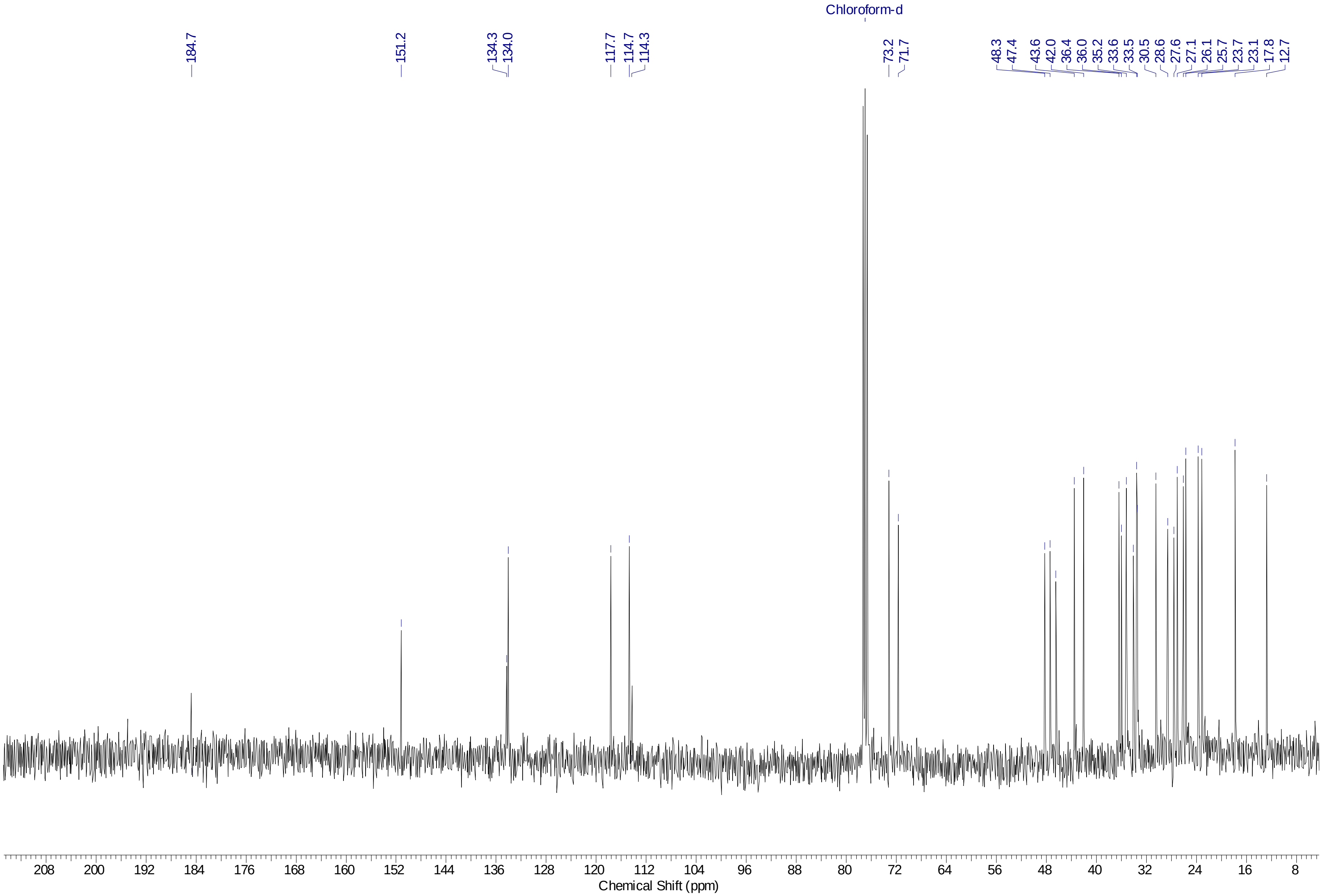

$^{1}$H NMR spectrum of 1,8-bis[(3α,5β,12α)-3,7,12-trihydroxycholan-24-ylamino]-9,10-anthraquinone (**5e**) ($CDCl_3$, 400 MHz, 300 K)

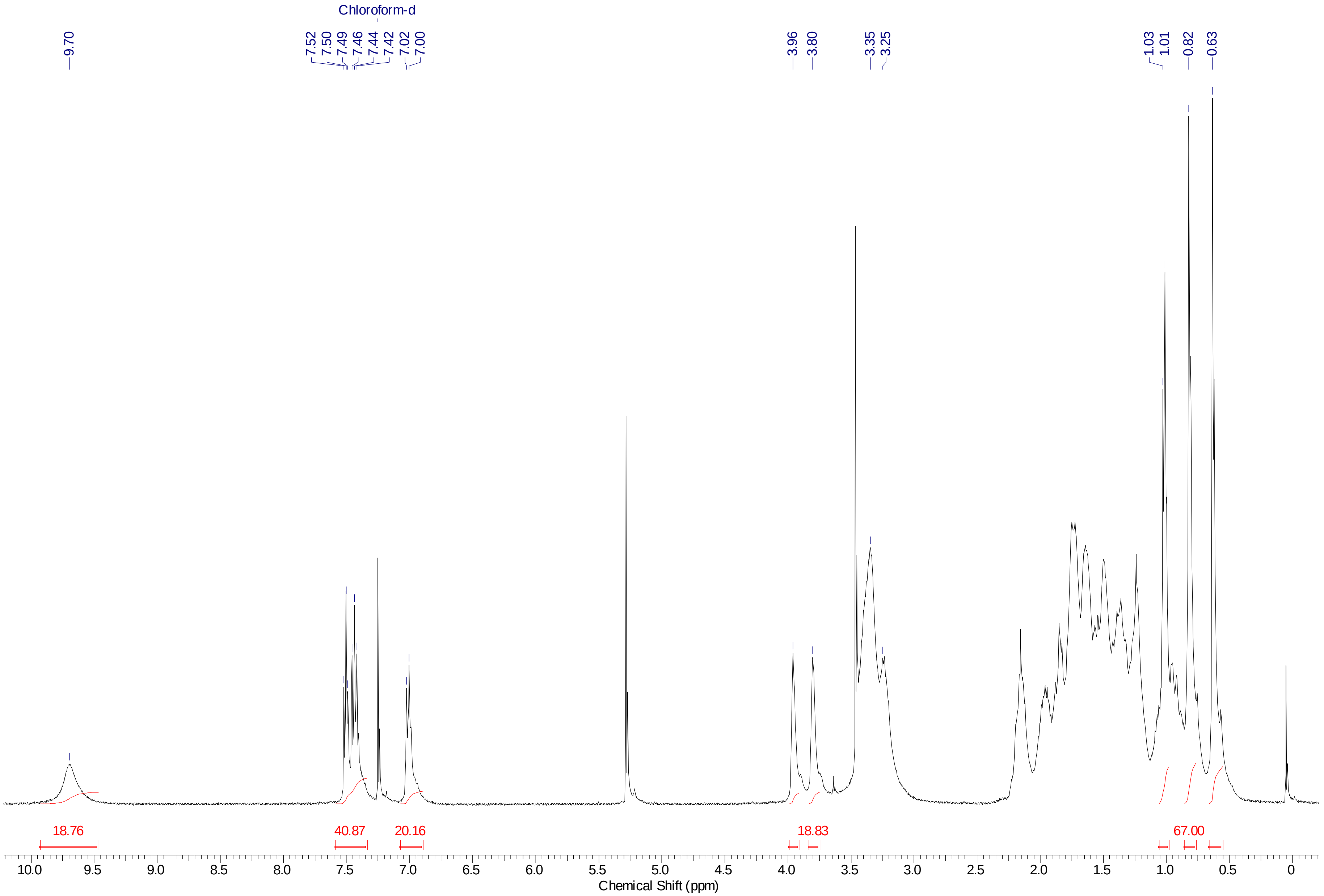

$^{13}C$ NMR spectrum of 1,8-bis[(3α,5β,12α)-3,7,12-trihydroxycholan-24-ylamino]-9,10-anthraquinone (**5e**) ($CDCl_3$, 100.6 MHz, 300 K)

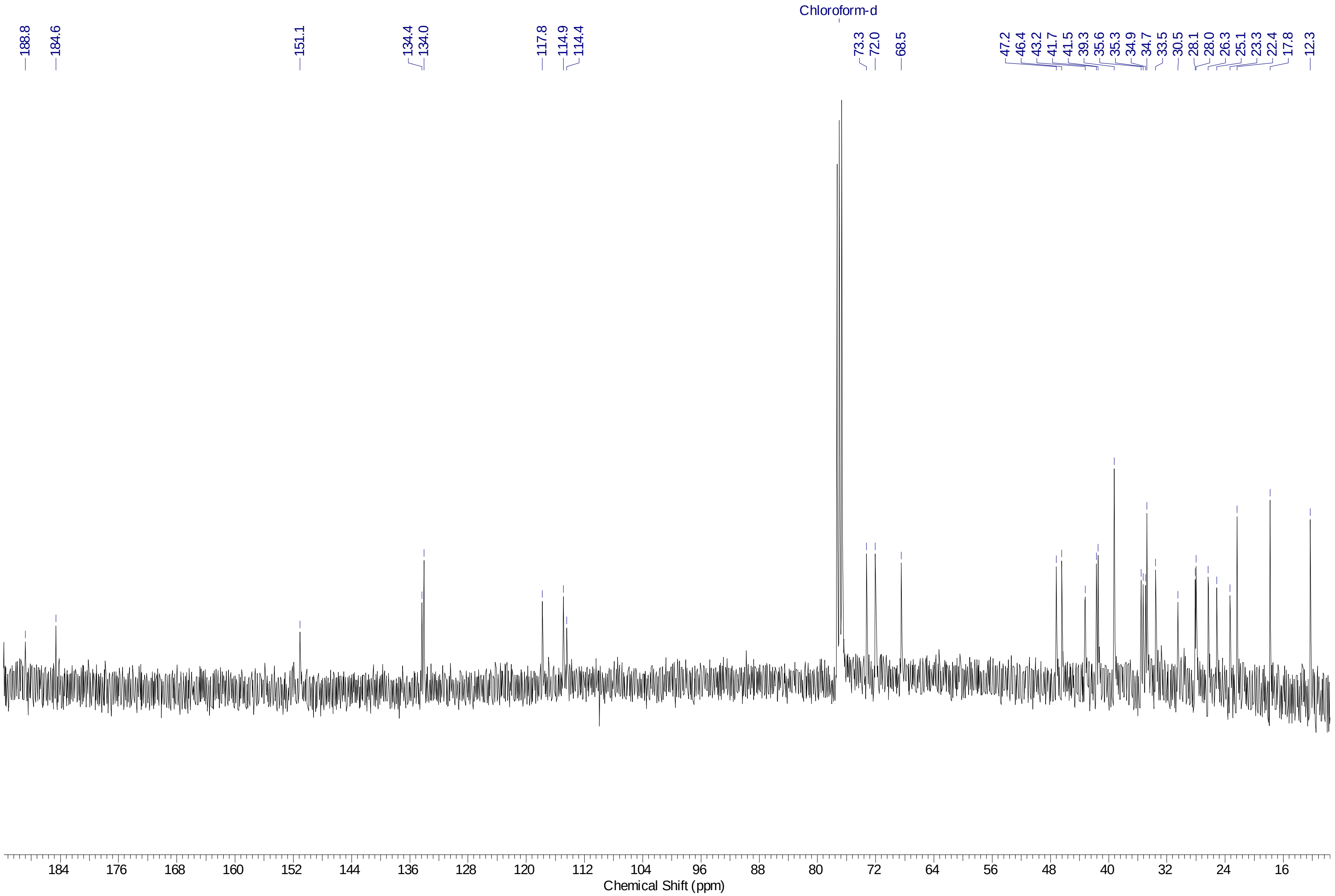

<sup></sup>

$^{1}$H NMR spectrum of 1,5-bis[(3α,5β)-3-hydroxycholan-24-ylamino]-9,10-anthraquinone (**5f**) ($CDCl_3$, 400 MHz, 300 K)

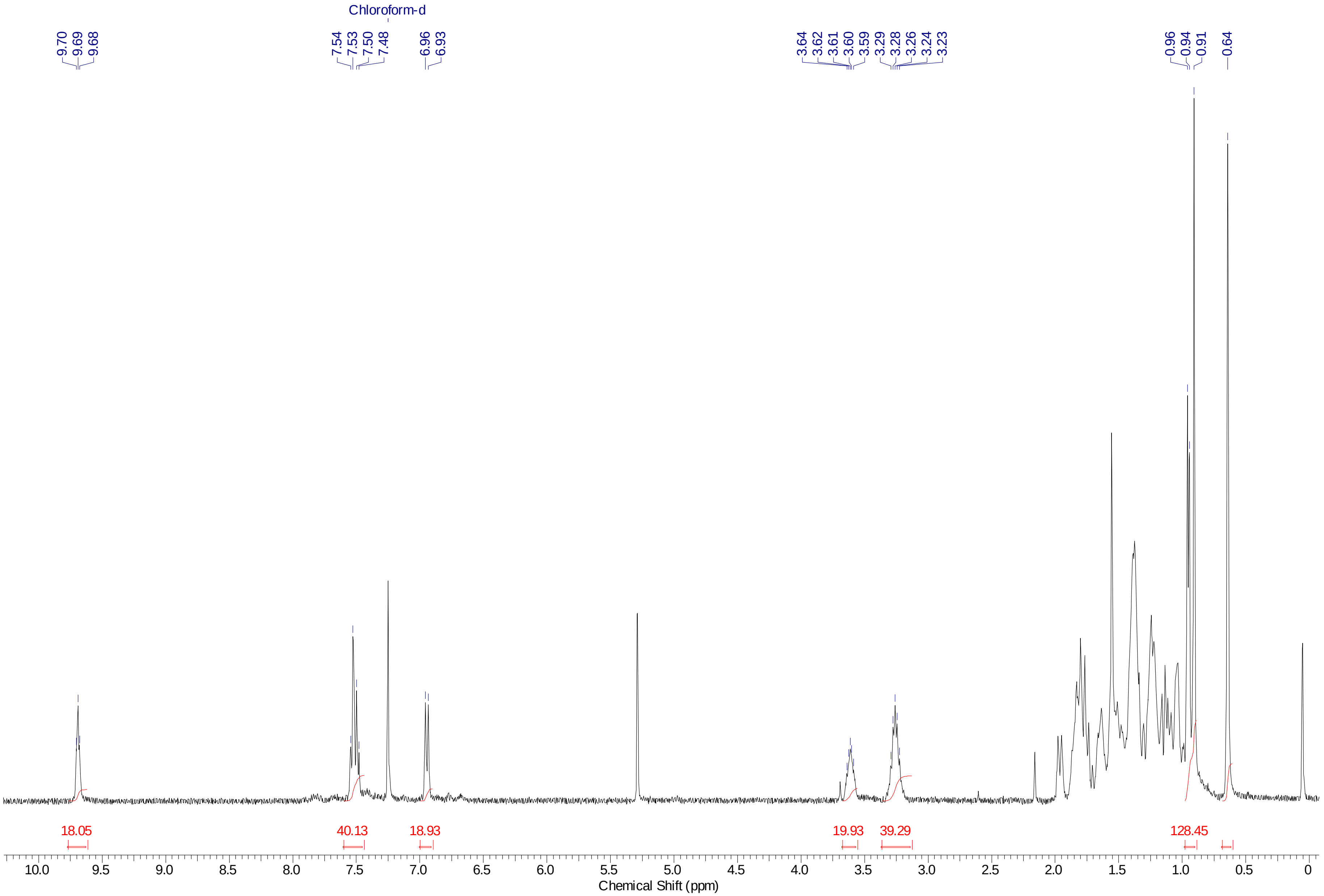

$^{13}$C NMR spectrum of 1,5-bis[(3α,5β)-3-hydroxycholan-24-ylamino]-9,10-anthraquinone (**5f**) ($CDCl_3$, 100.6 MHz, 300 K)

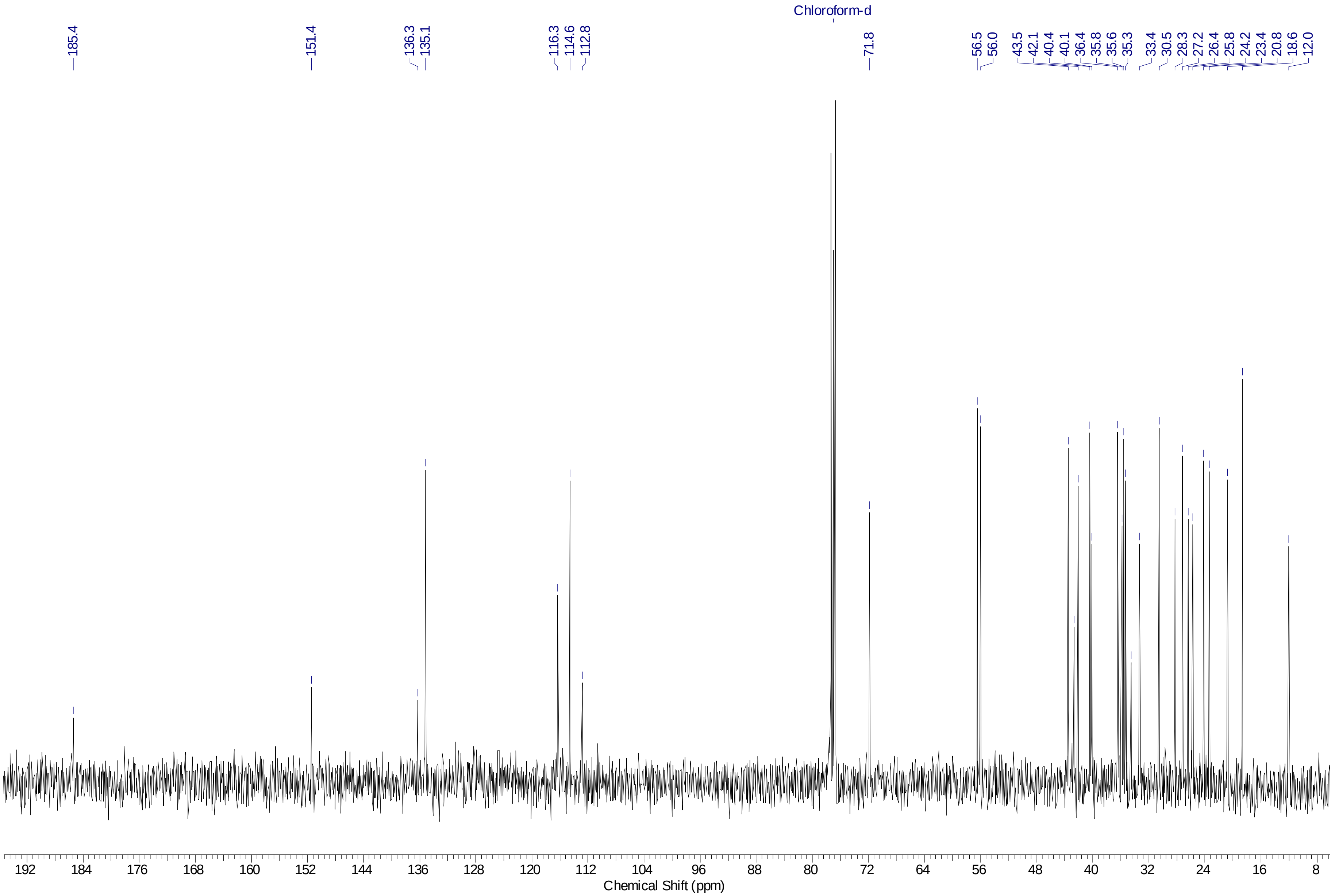

$^{1}$H NMR spectrum of 1,8-bis[(3α,5β)-3-methoxycholan-24-ylamino]-9,10-anthraquinone (**S1**) ($CDCl_3$, 400 MHz, 300 K)

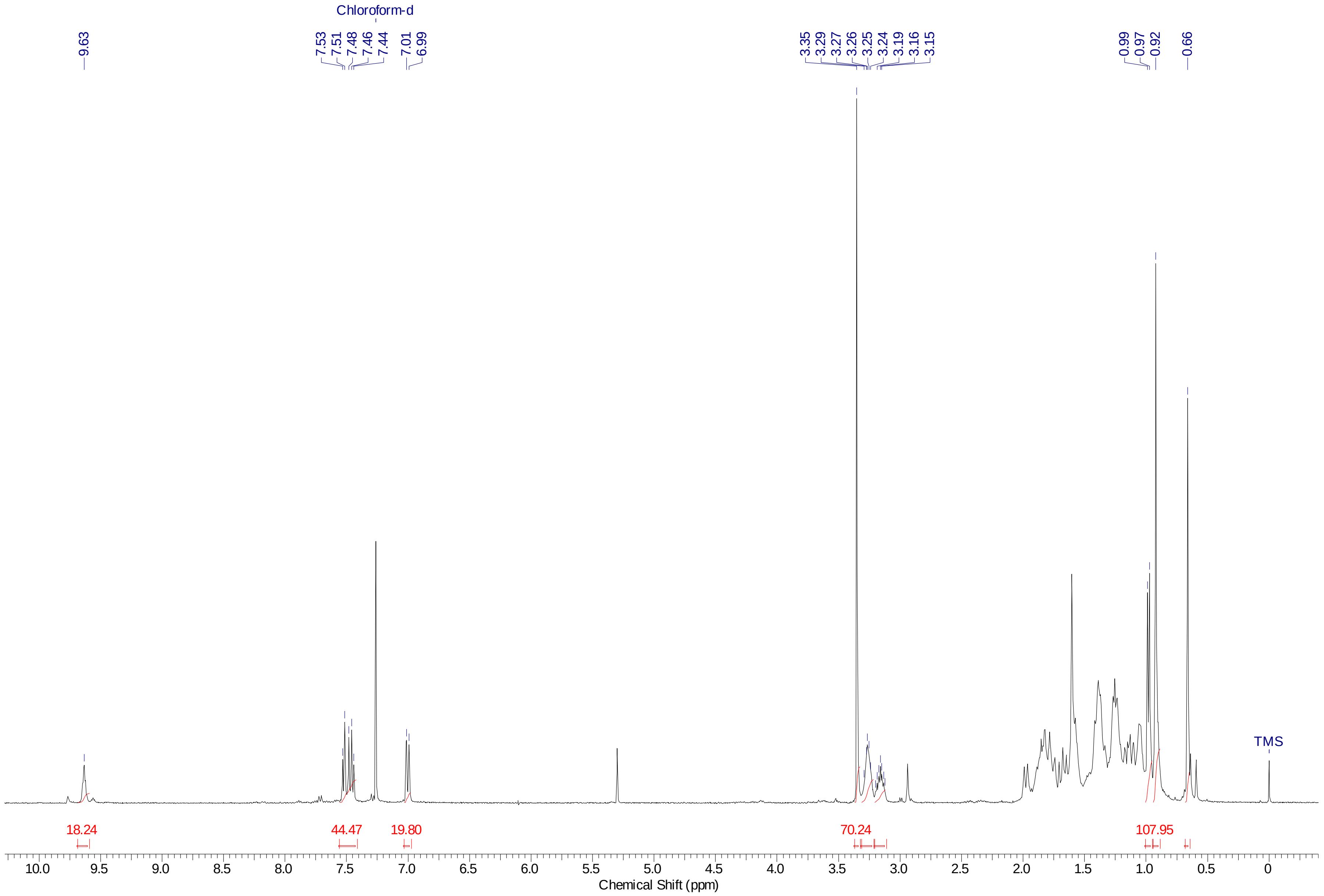

$^{13}$C NMR spectrum of 1,8-bis[(3α,5β)-3-methoxycholan-24-ylamino]-9,10-anthraquinone (**S1**) ($CDCl_3$, 100.6 MHz, 300 K)

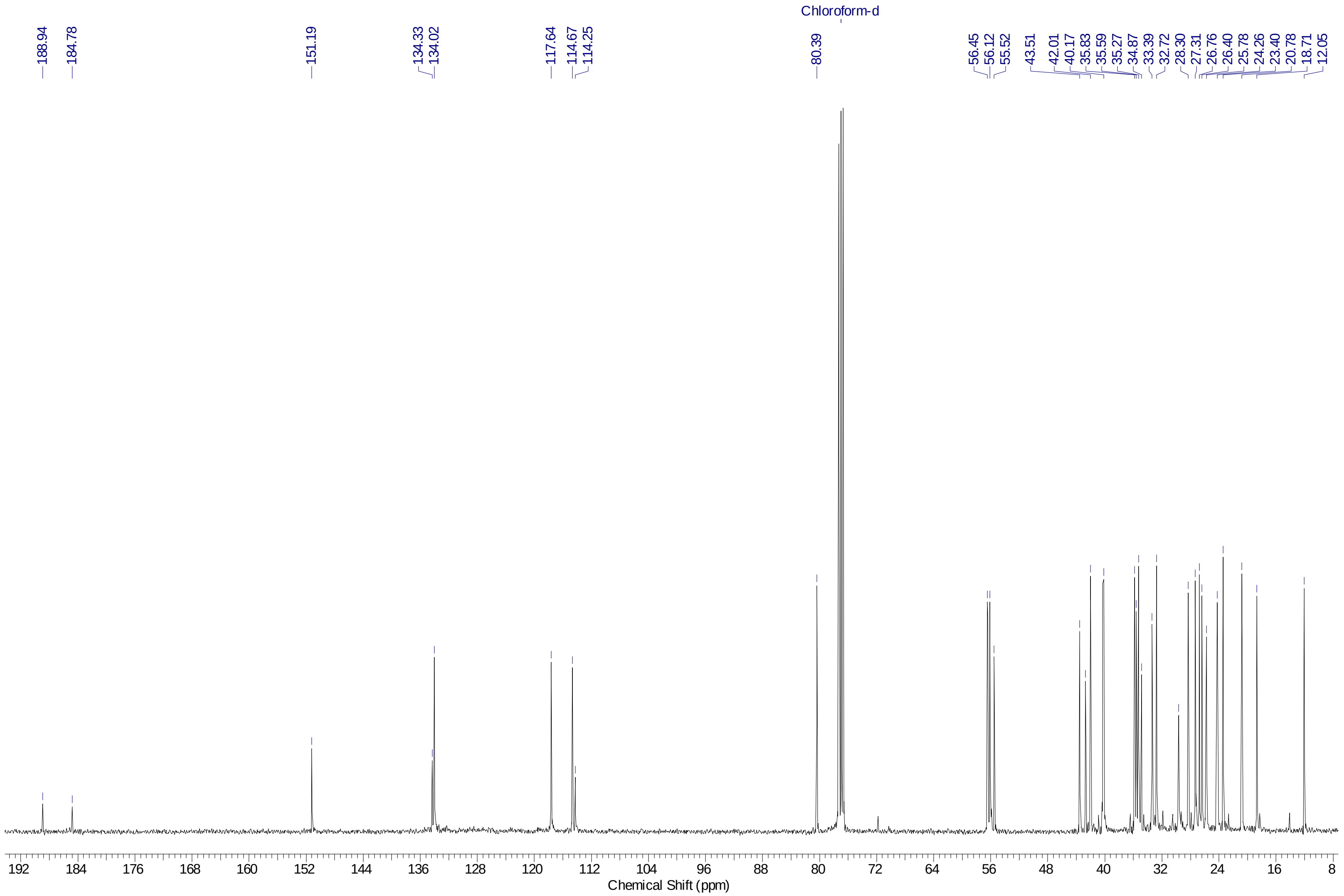

## UV–vis titration

**Figure S1:** UV–vis spectra of the ligand **5c** (50 µM solution in MeCN) before and after addition of 5 equiv of metal perchlorates

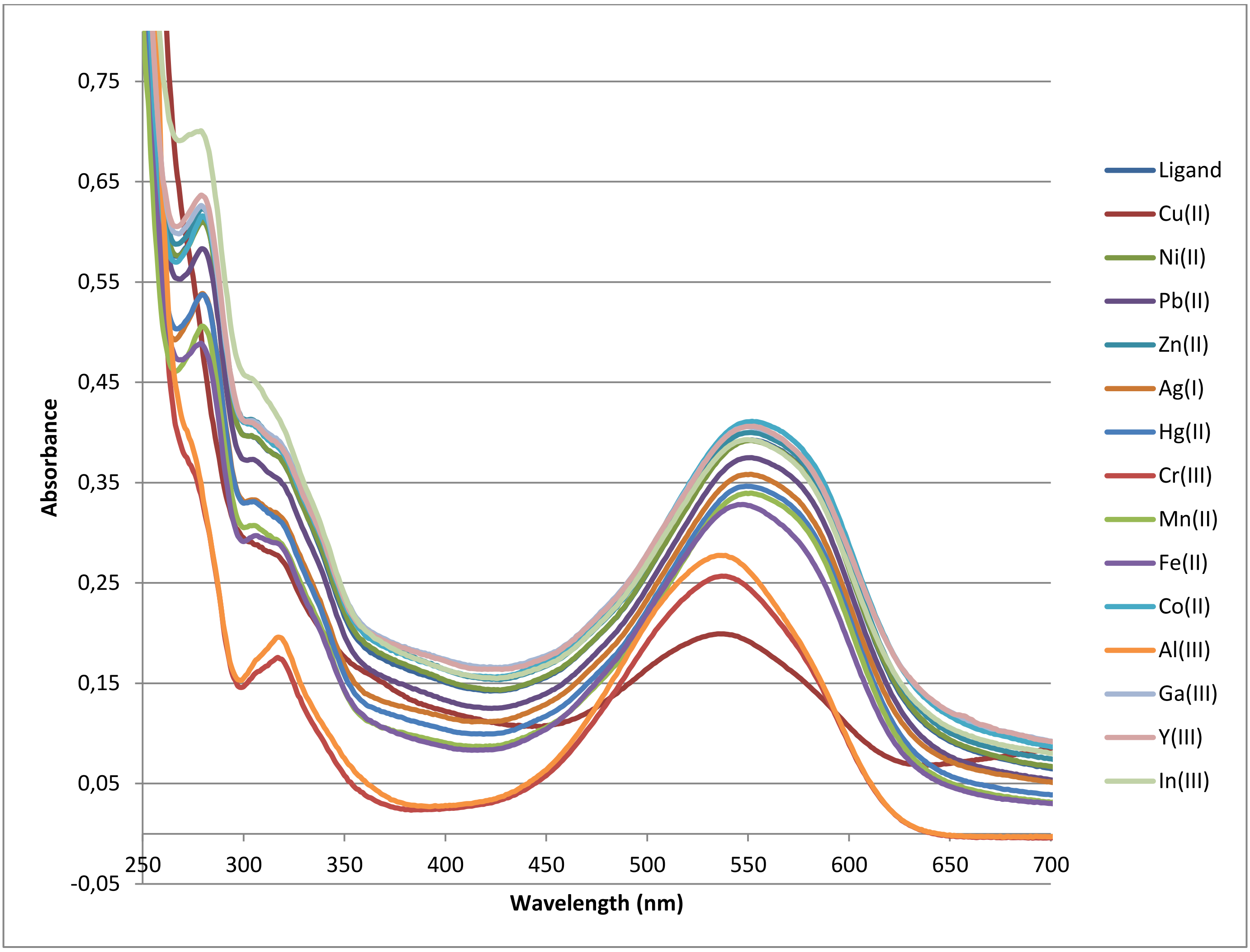

**Figure S2:** Evolution of UV–vis spectrum of **5c** (50 μM solution in MeCN) upon addition of $Cu(ClO_4)_2$ (0–5.0 equiv)

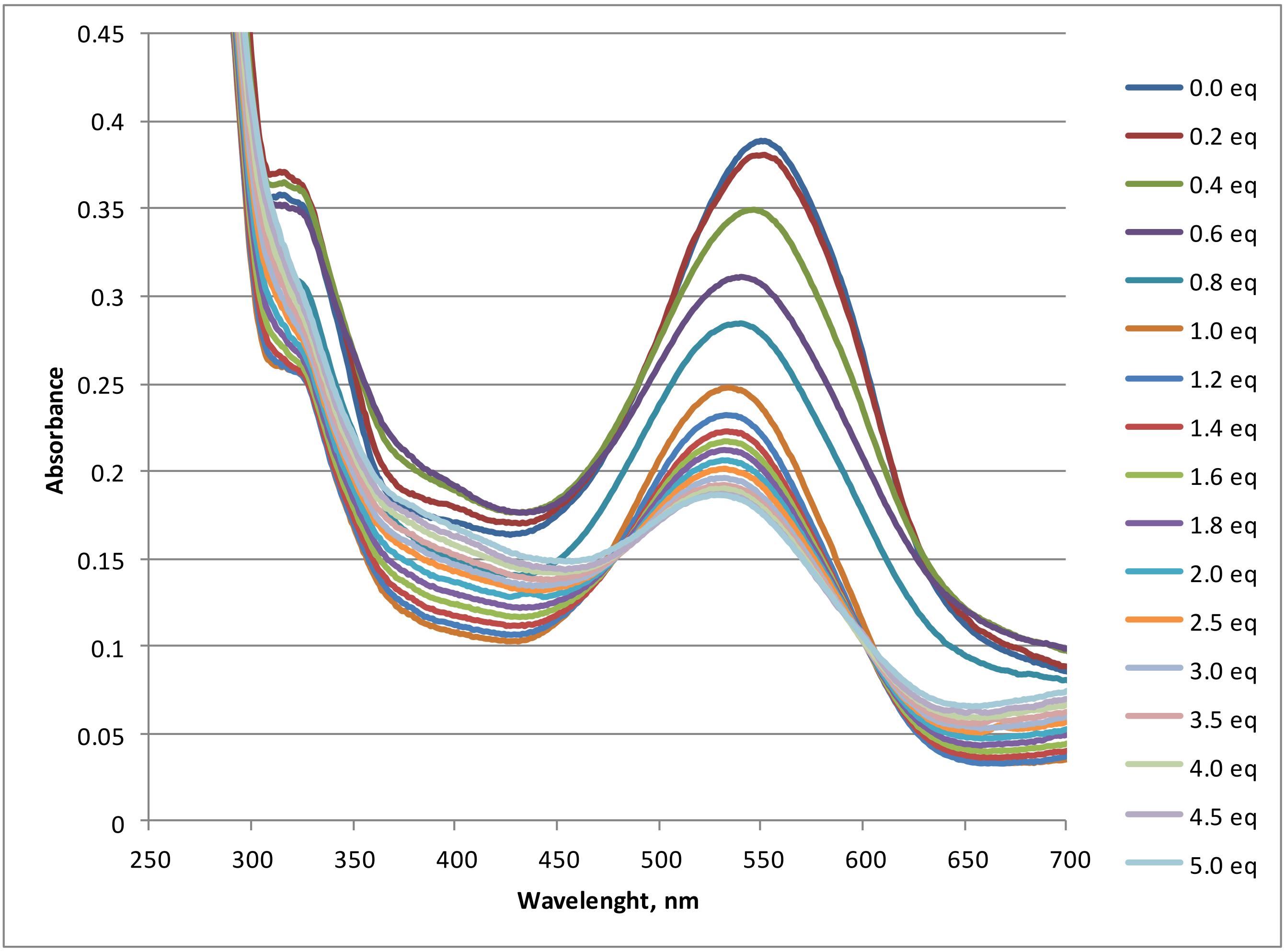


**Figure S3:** Changes of absorbance at 550 nm plotted against $[Cu(ClO_4)_2]$/[**5c**]$_{total}$

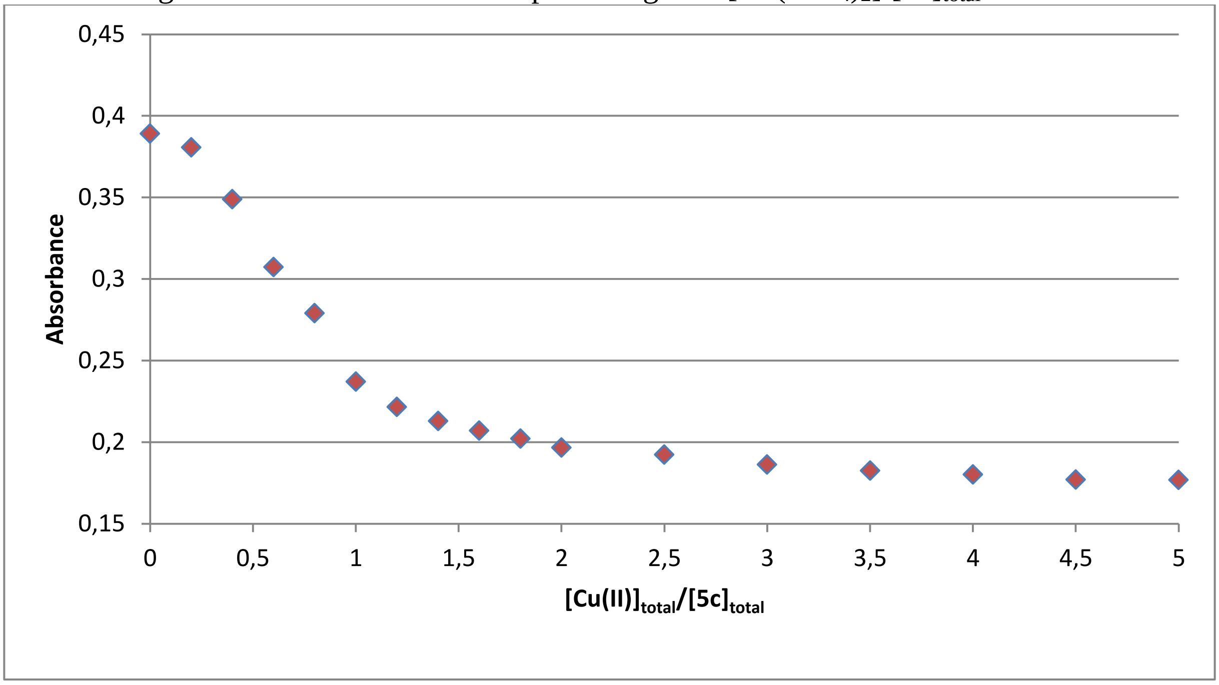

**Figure S4:** Evolution of UV–vis spectrum of **5c** (50 µM solution in MeCN) upon addition of $Al(ClO_4)_3$ (0–5.0 equiv)

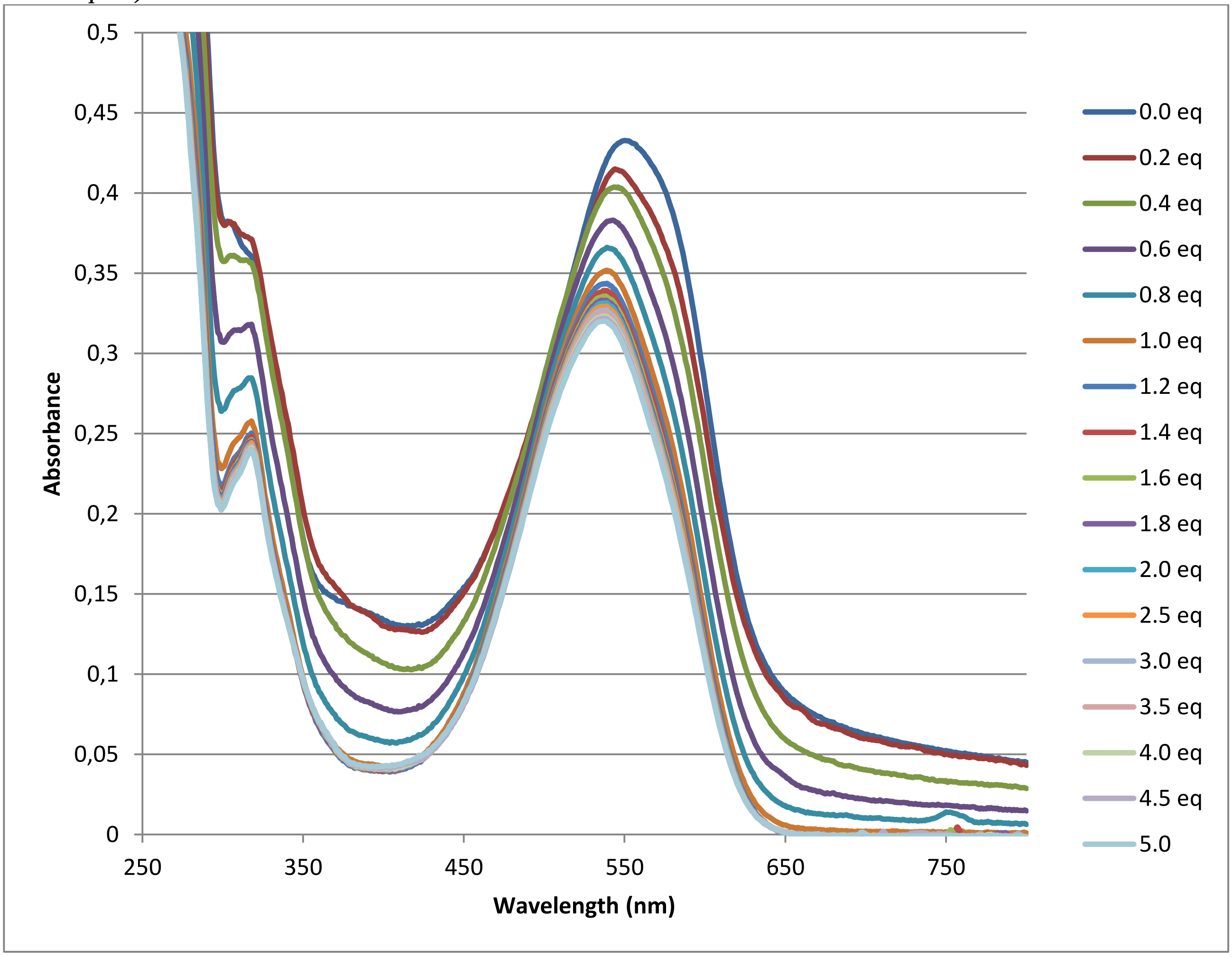


**Figure S5:** Changes of absorbance at 550 nm plotted against $[Al(ClO_4)_3]/[\mathbf{5c}]_{total}$

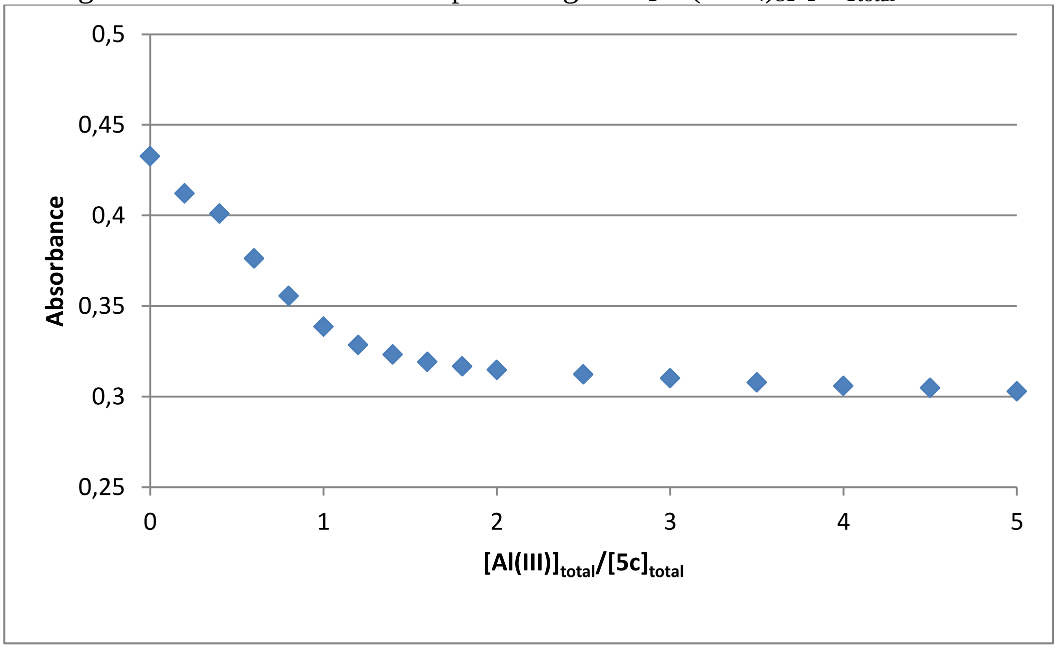

**Figure S6:** Evolution of UV–vis spectrum of **5c** (50 µM solution in MeCN) upon addition of $Cr(ClO_4)_3$ (0–5.0 equiv)

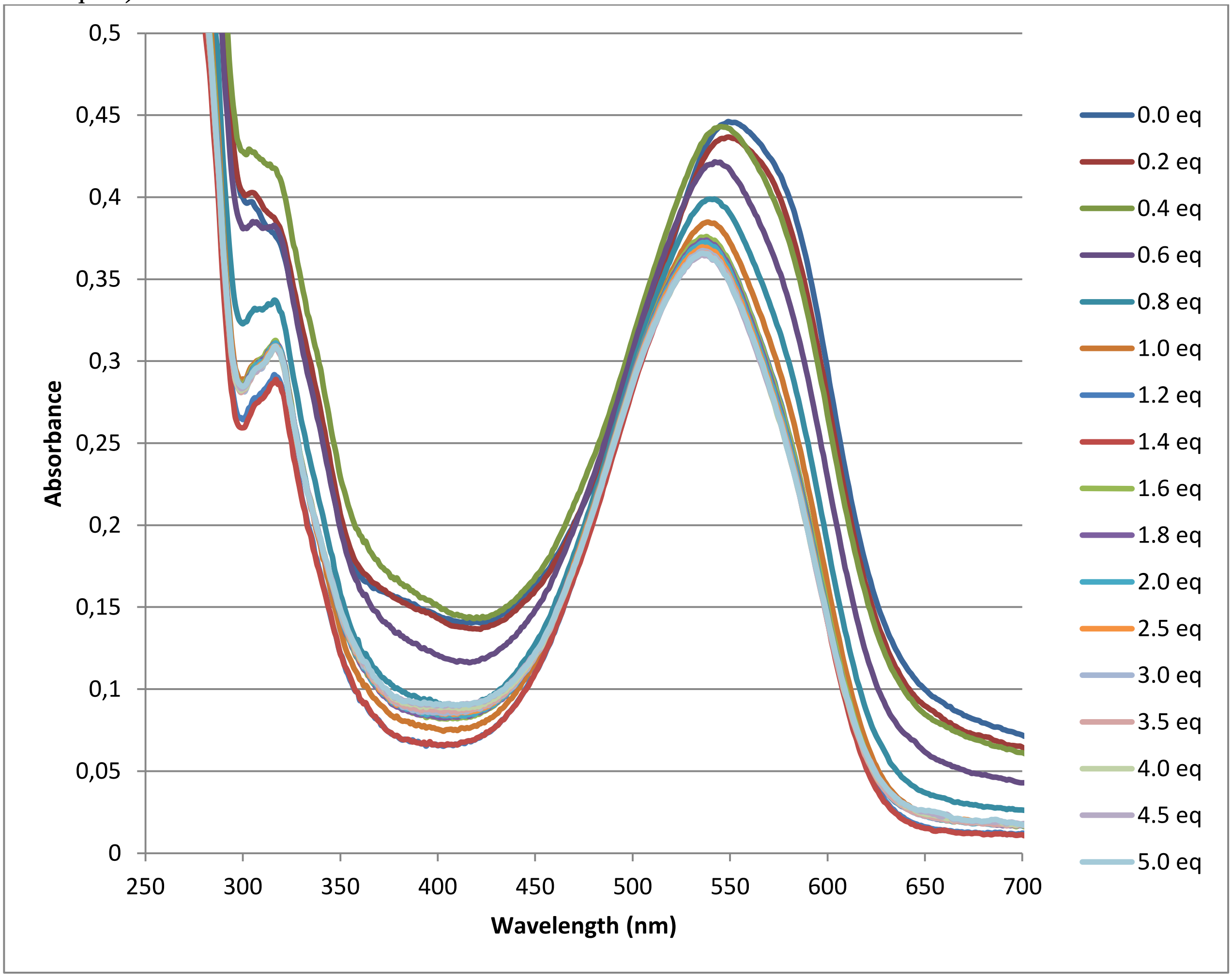


**Figure S7:** Changes of absorbance at 550 nm plotted against $[Cr(ClO_4)_3]/[\mathbf{5c}]_{total}$

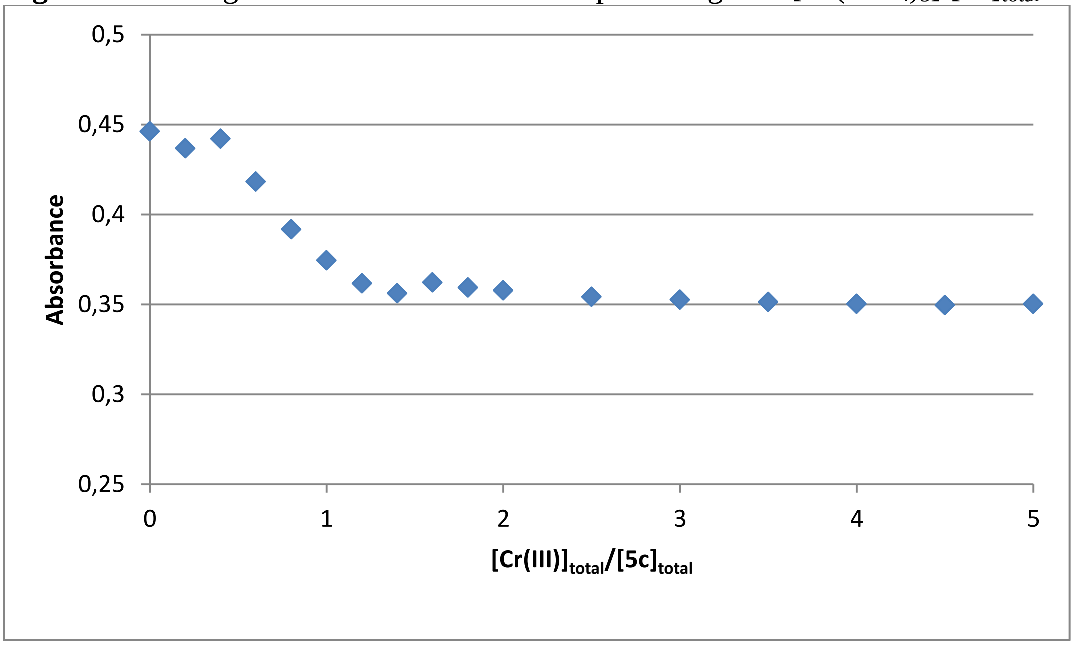

**Figure S8:** Evolution of UV–vis spectrum of **5d** (50 µM solution in MeCN) upon addition of $Cu(ClO_4)_2$ (0–5.0 equiv)

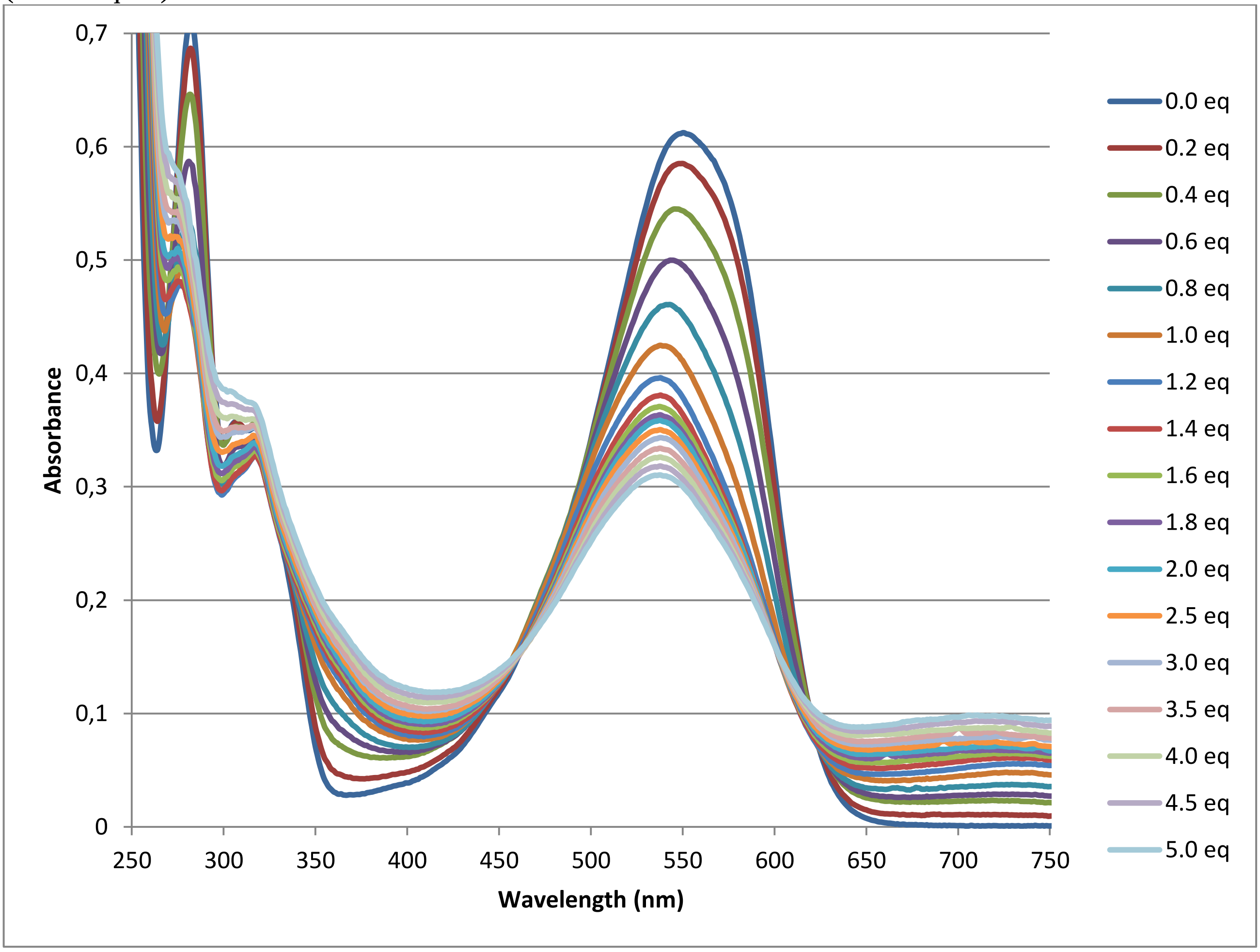


**Figure S9:** Changes of absorbance at 550 nm plotted against $[Cu(ClO_4)_2]$/[**5d**]$_{total}$

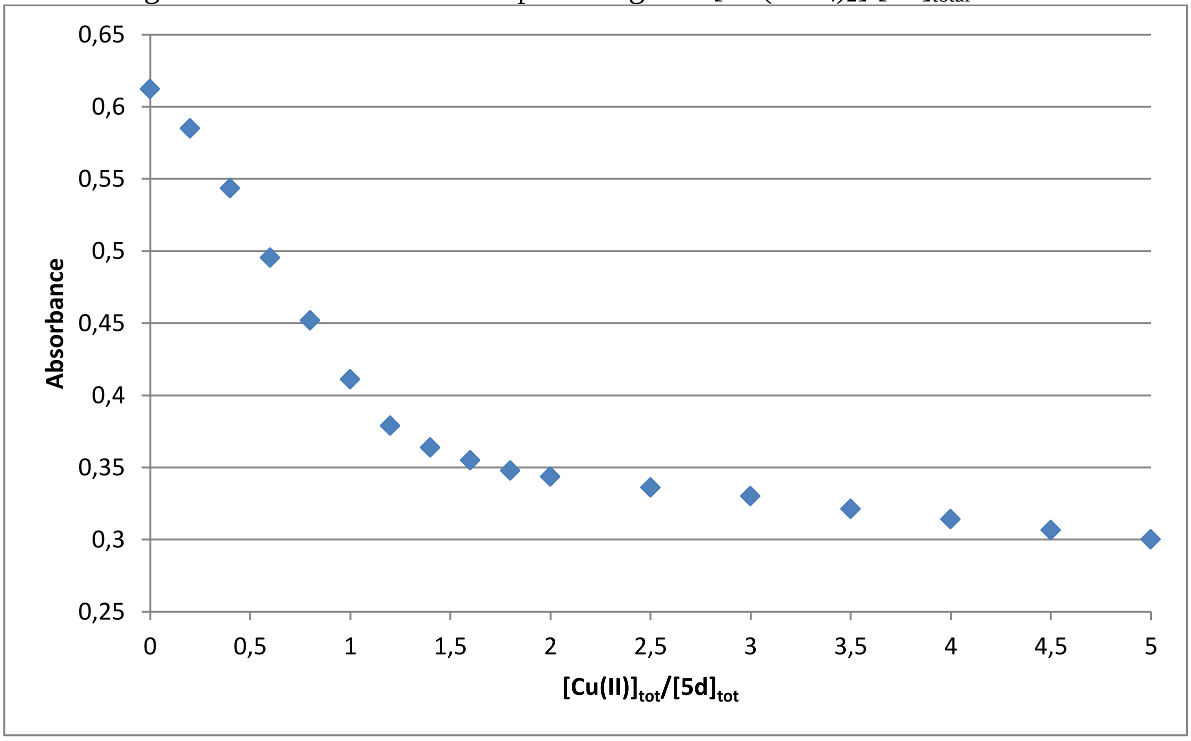

**Figure S10:** Evolution of UV–vis spectrum of **5d** (50 µM solution in MeCN) upon addition of $Al(ClO_4)_3$ (0–5.0 equiv)

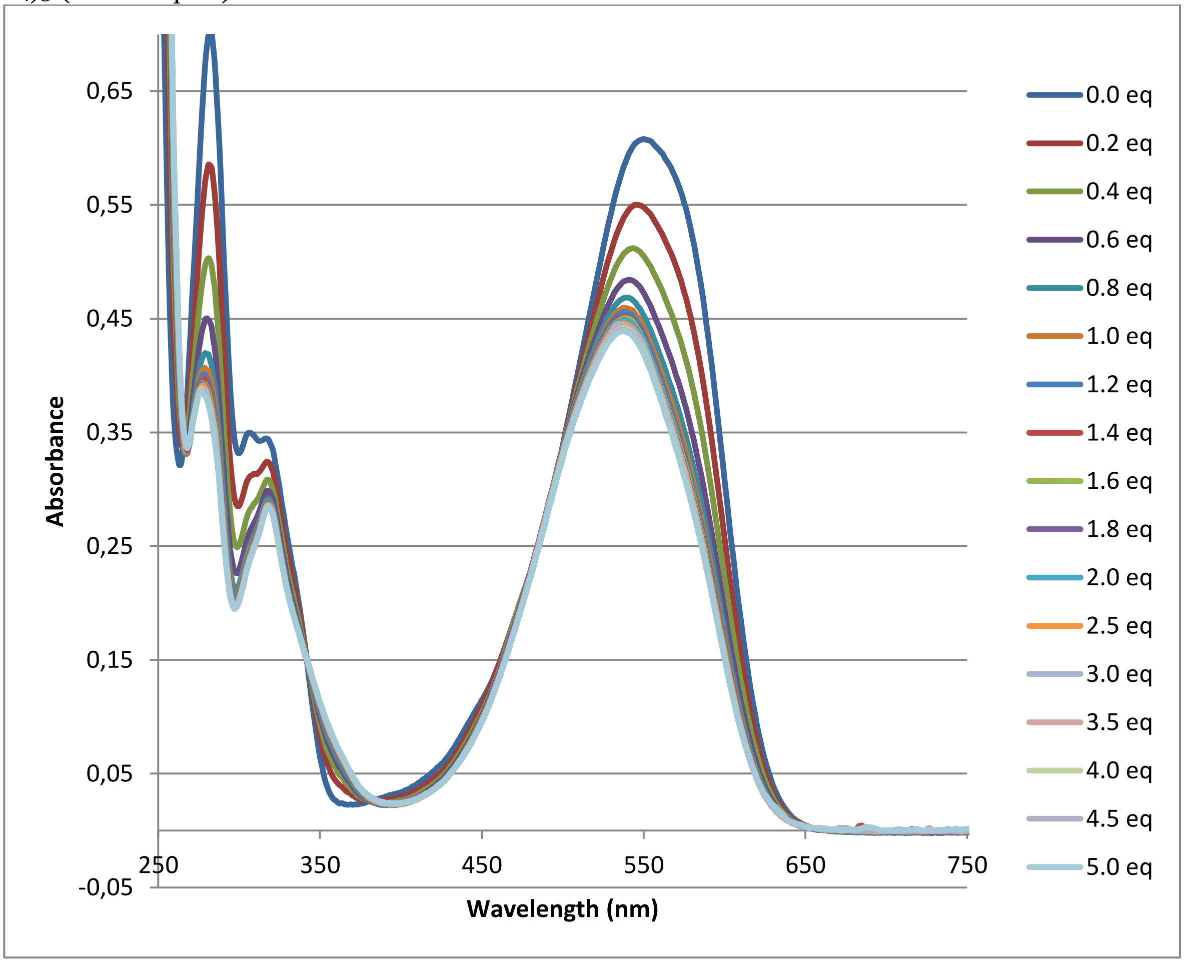


**Figure S11:** Changes of absorbance at 550 nm plotted against $[Al(ClO_4)_3]/[\mathbf{5d}]_{total}$

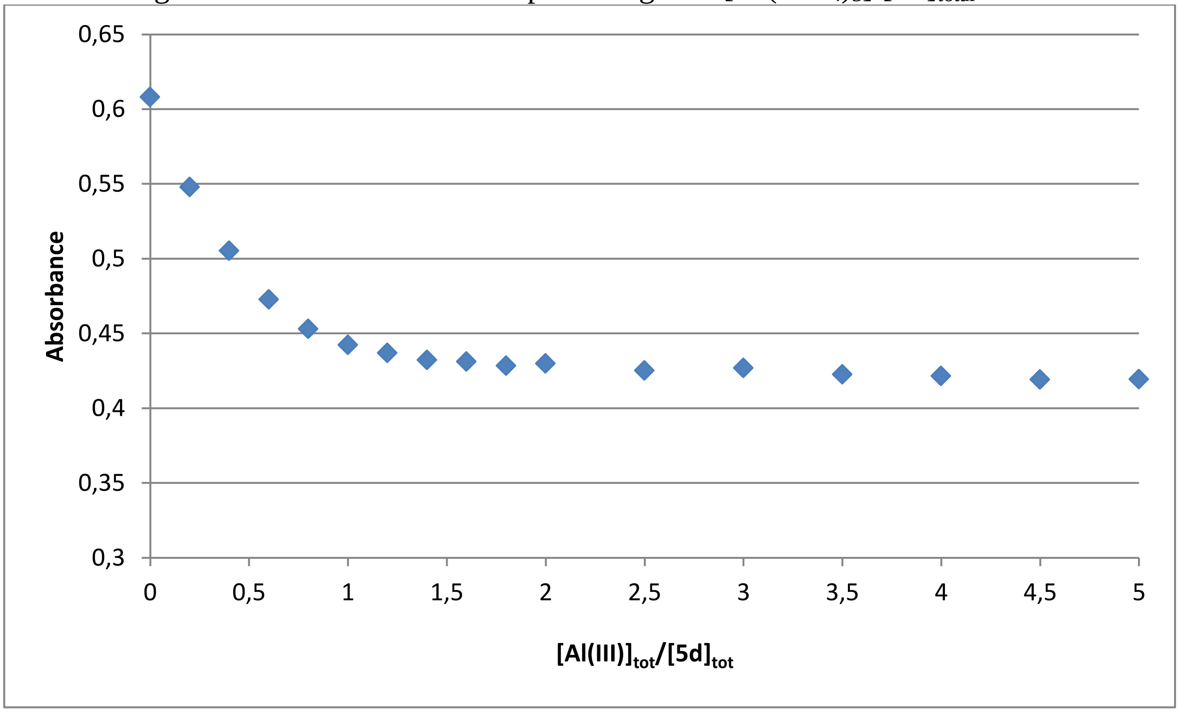

**Figure S12:** Evolution of UV–vis spectrum of **5d** (50 µM solution in MeCN) upon addition of $Cr(ClO_4)_3$ (0–5.0 equiv)

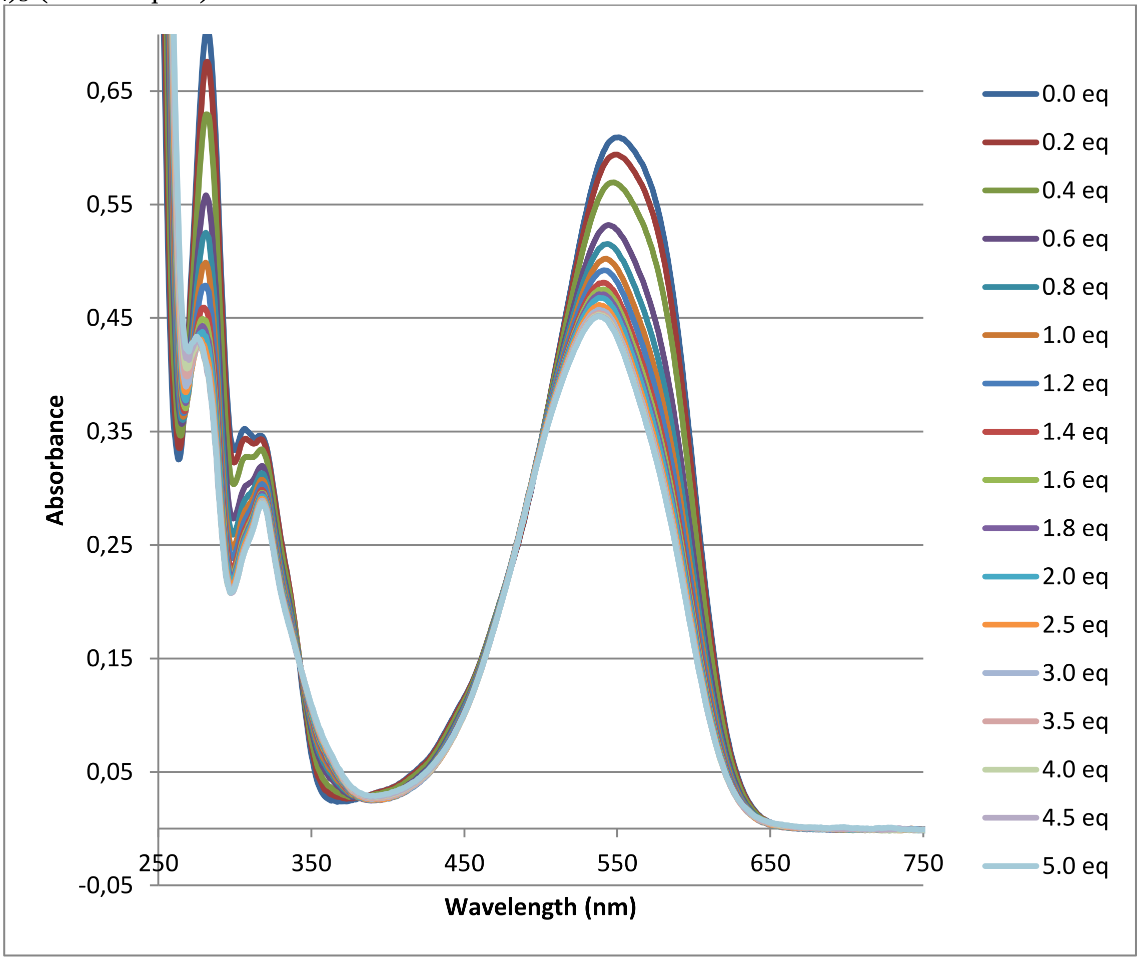


**Figure S13:** Changes of absorbance at 550 nm plotted against $[Cr(ClO_4)_3]/[\mathbf{5d}]_{total}$

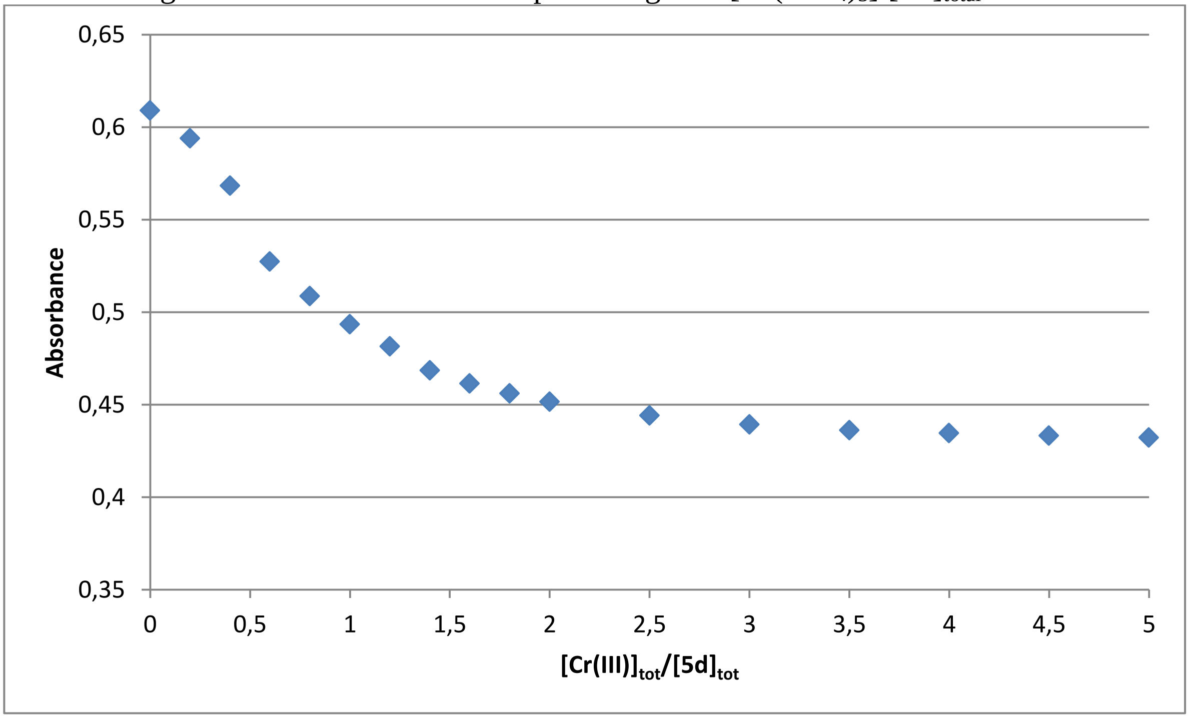

## References:


1. Binstead, R.A.; Jung, B.; Zuverbuhler, A.D. *SPECFIT/32, Global Analysis System, ver 3.0; Spectrum Software Associates: Marlborough, USA*, **2000**.

2. Fini, A.; Fazio, G.; Roda, A.; Bellini, A.M.; Mencini, E.; Guarneri, M. *J. Pharm Sci.* **1992**, *81*, 726-730.

3. Joachimiak, R.; Piasecka, M.; Paryzek, Z. *J. Chem. Res.*, **2008**, 260-265.
